# New insights into the neuroprotective and beta-secretase1 inhibitor profiles of tirandamycin B isolated from a newly found *Streptomyces composti* sp. nov.

**DOI:** 10.1038/s41598-023-32043-3

**Published:** 2023-03-24

**Authors:** Thitikorn Duangupama, Jaturong Pratuangdejkul, Sumet Chongruchiroj, Pattama Pittayakhajonwut, Chakapong Intaraudom, Sarin Tadtong, Patcharawee Nunthanavanit, Weerasak Samee, Ya-Wen He, Somboon Tanasupawat, Chitti Thawai

**Affiliations:** 1 Department of Biology, School of Science, King Mongkut’s Institute of Technology Ladkrabang, Bangkok, 10520 Thailand; 2 Department of Microbiology, Faculty of Pharmacy, Mahidol University, Phayathai, Bangkok, 10400 Thailand; 3 National Center for Genetic Engineering and Biotechnology (BIOTEC), National Science and Technology Development Agency (NSTDA), Thailand Science Park, Phaholyothin Road, Klong Luang, 12120 Pathum Thani Thailand; 4 Department of Pharmacognosy, Faculty of Pharmacy, Srinakharinwirot University, Nakhon Nayok, 26120 Thailand; 5 Department of Pharmaceutical Chemistry, Faculty of Pharmacy, Srinakharinwirot University, Nakhon Nayok, 26120 Thailand; 6 State Key Laboratory of Microbial Metabolism, School of Life Sciences & Biotechnology, Shanghai Jiao Tong University, Shanghai, 200240 People’s Republic of China; 7 Department of Biochemistry and Microbiology, Faculty of Pharmaceutical Sciences, Chulalongkorn University, Bangkok, 10330 Thailand; 8 Actinobacterial Research Unit, School of Science, King Mongkut’s Institute of Technology Ladkrabang, Bangkok, 10520 Thailand

**Keywords:** Biotechnology, Chemical biology, Microbiology

## Abstract

Tirandamycin (TAM B) is a tetramic acid antibiotic discovered to be active on a screen designed to find compounds with neuroprotective activity. The producing strain, SBST2-5^T^, is an actinobacterium that was isolated from wastewater treatment bio–sludge compost collected from Suphanburi province, Thailand. Taxonomic characterization based on a polyphasic approach indicates that strain SBST2-5^T^ is a member of the genus *Streptomyces* and shows low average nucleotide identity (ANI) (81.7%), average amino*-*acid identity (AAI) (78.5%), and digital DNA-DNA hybridization (dDDH) (25.9%) values to its closest relative, *Streptomyces thermoviolaceus* NBRC 13905^T^, values that are significantly below the suggested cut-off values for the species delineation, indicating that strain SBST2-5^T^ could be considered to represent a novel species of the genus *Streptomyces*. The analysis of secondary metabolites biosynthetic gene clusters (smBGCs) in its genome and chemical investigation led to the isolation of TAM B. Interestingly, TAM B at 20 µg/mL displayed a suppressive effect on beta-secretase 1 (BACE1) with 68.69 ± 8.84% inhibition. Molecular docking simulation reveals the interaction mechanism between TAM B and BACE1 that TAM B was buried in the pocket of BACE-1 by interacting with amino acids Thr231, Asp 228, Gln73, Lys 107 via hydrogen bond and Leu30, Tyr71, Phe108, Ile118 via hydrophobic interaction, indicating that TAM B represents a potential active BACE1 inhibitor. Moreover, TAM B can protect the neuron cells significantly (% neuron viability = 83.10 ± 9.83% and 112.72 ± 6.83%) from oxidative stress induced by serum deprivation and Aβ_1–42_ administration models at 1 ng/mL, respectively, without neurotoxicity on murine P19-derived neuron cells nor cytotoxicity against Vero cells. This study was reportedly the first study to show the neuroprotective and BACE1 inhibitory activities of TAM B.

## Introduction

Alzheimer’s disease (AD) is a common neurodegenerative disorder found in elderly people^1^. This disease is a common form of dementia, including loss of memory and cognitive function impairments^2,3^. The etiology of AD is unknown. However, several pathological hallmarks, oxidative stress, amyloid-β (Aβ) deposition, tau protein accumulation, and decreased levels of acetylcholine (ACh), show significant roles in the pathophysiology of this disease^2^. Nowadays, only the acetylcholinesterase inhibitors, rivastigmine, galantamine, donepezil, and a NMDA receptor antagonist, memantine, are approved by the US Food and Drug Administration (USFDA) for the treatment of AD^2^. Due to the complex nature of AD and these approved medications only delaying the progression of symptoms associated with AD, multi-targeting medication was needed to cure AD^1,2^. In addition, neuroprotection refers to mechanisms within the nervous system that relatively conserve the neuronal structure and/or function. Therefore, it is one of the strategies for treating neurodegenerative disorders, including AD^1^. The Aβ hypothesis is one of the Alzheimer’s pathological hallmarks. The Aβ, especially Aβ_42_, the oligopeptide with 42 amino acids involves in Alzheimer’s disease by generating oxidative stress in neuronal cells and inducing tau protein hyperphosphorylation resulting in toxicity in synapses and mitochondria. In addition, the aggregation of Aβ peptides leads to fibrils formation, which is lastly deposited as senile plaques^2^. The Aβ is derived from clavation of the amyloid precursor protein (APP) by beta-site APP cleaving enzyme-1 (BACE1, β-secretase) and γ-secretase. Thus, the inhibition of the proteolytic activity of BACE1 is interesting for Alzheimer’s disease treatment^2^. Natural products from microorganisms are a promising source for the pharmaceutical and biotechnological industries. Actinomycete, especially the genus *Streptomyces,* is known to be the largest taxon of the phylum *Actinomycetota* that have proven to be a rich source of secondary metabolites with diverse chemical structures and biological activities^4^. *Streptomyces* species are Gram-positive, filamentous actinobacteria that can produce both substrate and aerial mycelia, including spores directly on the aerial mycelium. The types of spore forms, e.g., straight to flexuous, verticillate, spiral, or open-loop, are commonly observed in this genus. All *Streptomyces* species are found to have *LL*-diaminopimelic acid in the peptidoglycan^5^. Many secondary metabolites isolated from *Streptomyces* spp. have been found to possess neuroprotective activity. For example, mescengricin from *Streptomyces griseoflavus* 2853-SVS4 could reduce the l-glutamate toxicity in chick primary mescencephalic neurons with an EC_50_ value of 6.0 nM^6^. Flaviogeranin, a 1,4-naphtoquinone derivative from *Streptomyces* sp. RAC226 possessed neuroprotective activity by preventing cell death in C6 cells treated with 100 nM of glutamate for 24 h with an EC_50_ of 8.6 nM^7^. Neuroprotectins A and B were isolated from *Streptomyces* sp. Q27107 and exhibited neuroprotective activity against the primary cultured chick telencephalic cells from glutamate- and kainite-induced neurotoxicities^8^. Lavanduquinocin is a carbazole derivative with an *ortho* quinone moiety isolated from the fermentation broth of *Streptomyces viridochromogenes*. This compound could reduce l-glutamate toxicity in neuronal hybridoma N18-RE-105 cells with EC_50_ values of 4.3 nM^9^. To date, only *N*-Methyl-D-Aspartate (NMDA) receptor antagonist drug (memantine), and AChE inhibitor drugs (Donepezil, and Galantamine) are approved for clinical treatment of AD^10–14^. Thus, an attempt to discover a drug candidate from new *Streptomyces* species habiting in unexamined environments is one strategy to discover the potential bioactive neuroprotective compounds from nature. Tirandamycins (TAMs), a group of tetramic acid antibiotic with a bicyclic ketal moiety, have been isolated from *Streptomyces* spp. Tirandamycins (TAMs) are generally known to be an antibacterial agents against Gram-positive and Gram-negative bacteria, including vancomycin-resistant enterococcus (VRE), and *Escherichia coli*^15,16^, with low cytotoxicity on human cells^17^. At the molecular level, TAMs are inhibitors of bacterial ribonucleic acid polymerase (RNAp) (TAM A, IC_50_ value of 0.8 mM)^18^. However, TAMs have never been reported to have neuroprotective properties. As part of our continuing work on the screening program for in vitro neuroprotective activity from secondary metabolites produced by *Streptomyces* species, we found that the crude extract from a culture broth of *Streptomyces* sp. SBST2-5^T^ exhibited an ability for neuronal protection by showing 110.70 ± 10.11% viability of neurons at the concentration of 1 ng/mL, suggesting the presence of compound(s) with the ability to prevent neuron cell death and led to the discovery of TAM B as the major component involved in this neuroprotective activity. In this study, we have additionally used molecular docking to understand the molecular mechanism of the BACE1 inhibition by TAM B. This is the first report on the mechanism of action of TAM B through anti-BACE1 and neuroprotective activities. The knowledge obtained in this study would be helpful in the rational design of TAM derivatives to be more effective in binding to the BACE1 and be essential in exploring TAM derivatives as lead compounds among natural neuroprotective and anti-BACE1 drugs for the treatment of AD. Besides, the genome-based taxonomic characterization of *Streptomyces* sp. SBST2-5^T^, the isolation and identification of the isolated compounds, the evaluation of anti-acetylcholinesterase (anti-AChE), anti-beta-secretase (anti-BACE1), anti-oxidative, neuroprotective, and cytotoxicity activities are also reported.

## Results and discussion

### Polyphasic taxonomic characterizations of strain SBST2-5^T^

**Figure 1:**
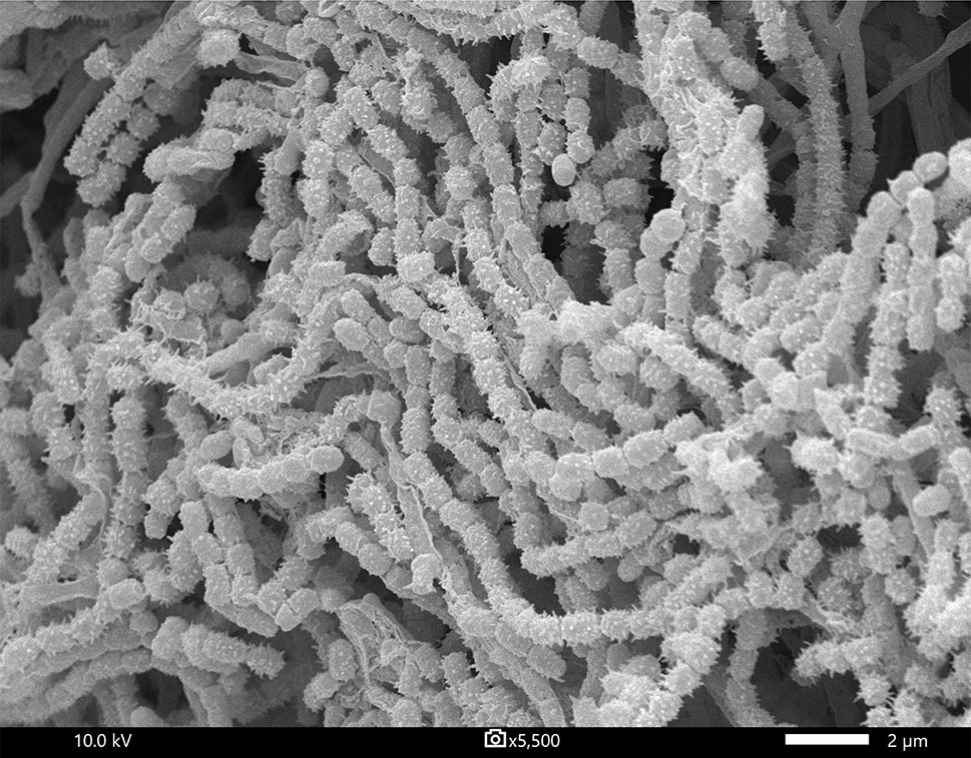
Scanning electron micrograph of strain SBST2-5^T^ grown on ISP 2 agar for 14 days at 30 °C. Bar, 2 μm.

**Figure 2:**
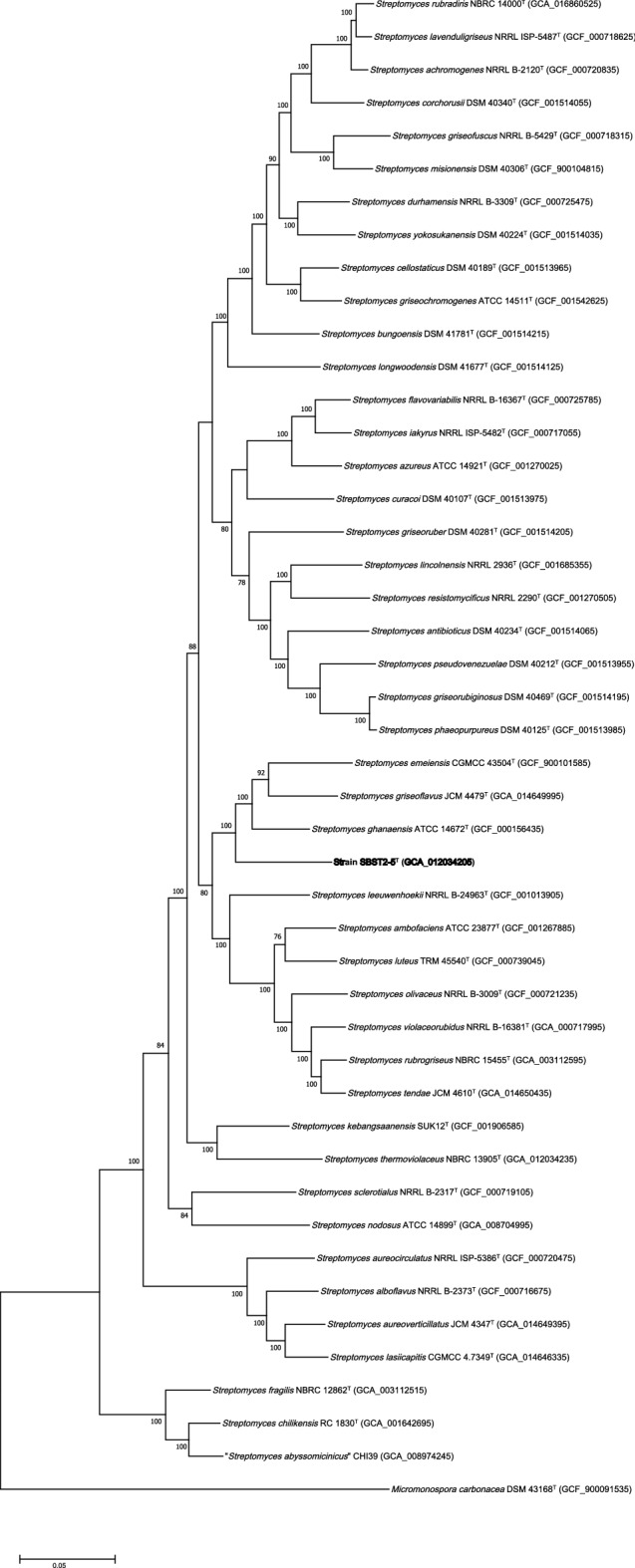
Phylogenomic analysis of strain SBST2-5^T^ and type strains affiliated to the genus *Streptomyces* based on 120 bacterial conserved single copied gene sets of the members. The bootstrap values on the nodes are displayed by > 50. Bar, 0.05 represents the nucleotide substitution per position.

Investigating novel actinomycete from unexamined environments is one strategy to discover promising bioactive compounds from microbial resources. Wastewater treatment is a process used to remove contaminants from wastewater and convert it into an effluent that can be returned to the water cycle. During the wastewater treatment process, the environment inside the system has been found to have a high temperature and less oxygen content. Thus, actinomycetes living in the wastewater treatment process are expected to be different from other terrestrial actinomycetes. An actinobacterium, designed strain SBST2-5^T^, was isolated from the wastewater treatment bio–sludge compost collected from Suphanburi province, Thailand. Strain SBST2-5^T^ grew easily on the International *Streptomyces* Project 2 medium (ISP 2). The mature aerial spore mass is grayish white on the culture media tested after 14 days of cultivation at 30 °C (Additional file: Fig. S1). Analysis of whole-cell hydrolysates of strain SBST2-5^T^ revealed the presence of *LL*-diaminopimelic acid. Galactose, glucose, mannose, and ribose were detected as whole-cell sugars (Additional file: Fig. S2). It contained MK-9(H_6_) and MK-9(H_8_) as predominant menaquinones while MK-9(H_4_) was minor component. Diphosphatidylglycerol, phosphatidylethanolamine, phosphatidylglycerol, phosphatidylinositol, phosphatidylinositol mannoside, three unidentified phospholipids, and ninhydrin-positive lipid were detected (Additional file: Fig. S3). The predominant fatty acids (> 10%) were observed to be *iso*-C_15:0_ (27.4%), *iso*-C_16:0_ (23.0%), and *anteiso*-C_15:0_ (15.7%) (Additional file: Table S1). This major fatty acid pattern was also found in *Streptomyces thermoviolaceus* NBRC 13905^T^ with different proportions. Scanning electron microscopy shows that the isolate forms a long straight chain of spores with a hairy surface (Fig. 1). The morphological and chemotaxonomic properties of the isolate are consistent with its classification in the genus *Streptomyces*^19^. To identify the taxonomic status at the species level, we first analyzed the 16S rRNA gene sequence (1430 nt) of strain SBST2-5^T^, and the sequence was submitted to Genbank with the accession number LC430996. The result showed that strain SBST2-5^T^ is a member of the genus *Streptomyces,* with *S. thermoviolaceus* NBRC 13905^T^ being the closest neighbor exhibiting the highest 16S rRNA gene sequence similarity value of 98.9% to strain SBST2-5^T^, slightly higher than the 98.6% proposed threshold for species classification, as recommended by Kim et al.^20^. Recently genome-based taxonomic approaches such as ANI (< 95%)^21^, AAI (< 95–96%)^22^, and dDDH (< 70%)^23,24^ have been used as the threshold recommended for differentiating strains into different species; the values in parentheses are cut-off values. Strain SBST2-5^T^ was found to have the ANIb (81.7–84.9%), AAI (78.5–82.8%), and dDDH (25.9–30.0%) values that were significantly lower than the above cut-off values for species demarcation, indicating that the strain may represent a new species of the genus *Streptomyces* (Additional file: Table S2). In addition, the taxonomic position of strain SBST2-5^T^ in the 16S rRNA gene tree (ML tree) indicated that the strain was clustered in the genus *Streptomyces* and was placed in a different species node from *S. thermoviolaceus* NBRC 13905^T^, the closest relatives (Additional files: Fig. S4). In contrast, the position of strain SBST2-5^T^ in the NJ tree formed a clade together with *S. thermoviolaceus* NBRC 13905^T^ (Additional file: Fig. S5). The genome-based phylogenetic tree reconstruction by automatic selection of the most closely related type strains by autoMLST revealed that strain SBST2-5^T^ was positioned with *Streptomyces emeiensis* CGMCC 4.3504^T^^25^, *Streptomyces griseoflavus* JCM 4479^T^^26^, and *Streptomyces ghanaensis* ATCC 14672^T^^27,28^. This result emphasized that the position of strain SBST2-5^T^ was separated from the branches carrying *S. thermoviolaceus* NBRC 13905^T^ (Fig. 2). Moreover, the strain could be distinguished from its closest related type strains, *S. thermoviolaceus* NBRC 13905^T^, *S. emeiensis* CGMCC 4.3504^T^, *S. griseoflavus* JCM 4479^T^, and *S. ghanaensis* ATCC 14672^T^ by its physiological and biochemical characteristics (Additional file: Table S3). Unlike *S. thermoviolaceus* NBRC 13905^T^, strain SBST2-5^T^ could utilize l-arabinose, d-cellobiose, d-galactose, *myo*-inositol, d-mannose, d-melibiose, d-raffinose, sucrose, d-trehalose, and d-xylitol as sole carbon sources and showed positive results for nitrate reduction, and decomposition of hypoxanthine but the closest related type strain could not. Additionally, strain SBST2-5^T^ tolerates NaCl up to 6%, but *S. thermoviolaceus* NBRC 13905^T^ could only tolerate NaCl up to 1% (w/v). In addition, compared to strain SBST2-5^T^, *S. emeiensis* CGMCC 4.3504^T^ was not able to use d-galactose, d-raffinose, sucrose as sole carbon sources and could not grow at 55 °C, while *S. griseoflavus* JCM 4479^T^ did not use l-arabinose, d-galactose, d-raffinose, sucrose as sole carbon sources. Moreover, the ability to utilize l-arabinose, d-raffinose, sucrose, dl-2-aminobutyric acid, l-cysteine, l-methionine, and the production of α-mannosidase, trypsin were significant phenotypic differences between strain strain SBST2-5^T^ and *S. ghanaensis* ATCC 14672^T^. It is evident from taxonomic data that strain SBST2-5^T^ could be judged to represent a novel species of the genus *Streptomyces*, for which the name *Streptomyces composti* sp. nov. is proposed.

### Genome analysis for secondary metabolites of strain SBST2-5^T^

Members of the genus *Streptomyces* are a vital microbial resource because of their potential to synthesize many bioactive natural products with therapeutic applications^29^. Since *Streptomyces* spp. have an essential capacity to produce valuable secondary metabolites, exploring new taxa is one strategy that leads to discovering new secondary metabolites from nature. To date, a bioinformatic tool such as antiSMASH^30^ has been used to describe and/or identify the biosynthesis gene clusters (BGCs) in the genome of the actinobacteria. It also could be used for addressing the natural product potential of *Streptomyces* spp. The draft genome *Streptomyces* sp. strain SBST2-5^T^ is composed of 100 contigs with an N50 value of 166 kb. The total genome size is about 6.5 Mb, with genome coverage of 200X. The digital G+C content is 72.2%, indicating in the range of the genus *Streptomyces*^19^ (Additional file: Table S4). It was deposited in GenBank under the accession number JAATEM000000000. We used antiSMASH version 7.0 beta^30^ to preliminarily determine and compare the putative biosynthetic gene clusters in the genome of strain SBST2-5^T^. It was found that the genome of strain SBST2-5^T^ was found to be rich in terpene and NRPS-independent-siderophore clusters. One of them showed 86% homology to the tirandamycin (*tam*) biosynthetic gene cluster in *Streptomyces* sp. 307–9^31^ (Additional files: Fig. S6 and Table S5). A detailed analysis of the detected cluster in *Streptomyces* sp. SBST2-5^T^ showed its difference from the described *tam* cluster from *Streptomyces* sp. 307–9. However, the key tirandamycin-forming genes involved in core biosynthetic genes (open reading frame (Orf) 2 and Orf 6), additional biosynthetic genes (Orf3 and Orf12), transport-related gene (Orf13), regulatory genes (Orf11 and Orf15), and other genes (Orf5 and Orf7-9) were observed. In addition, additional biosynthetic genes (Orf1, Orf17, and Orf19) and other genes (Orf4, Orf10, Orf14, Orf16, and Orf18) were observed between the tirandamycin-forming genes in the cluster from the SBST2-5^T^ strain. (Additional files: Fig. S7 and Table S6). According to the taxogenomic features and the bioinformatic analysis of the gene clusters led to the discovery of TAM and represented the strain as a new TAM producer. It can be concluded that strain SBST2-5^T^ is a potentially prolific source of TAM.

### Compounds isolated from *Streptomyces* sp. SBST2-5^T^

**Figure 3:**
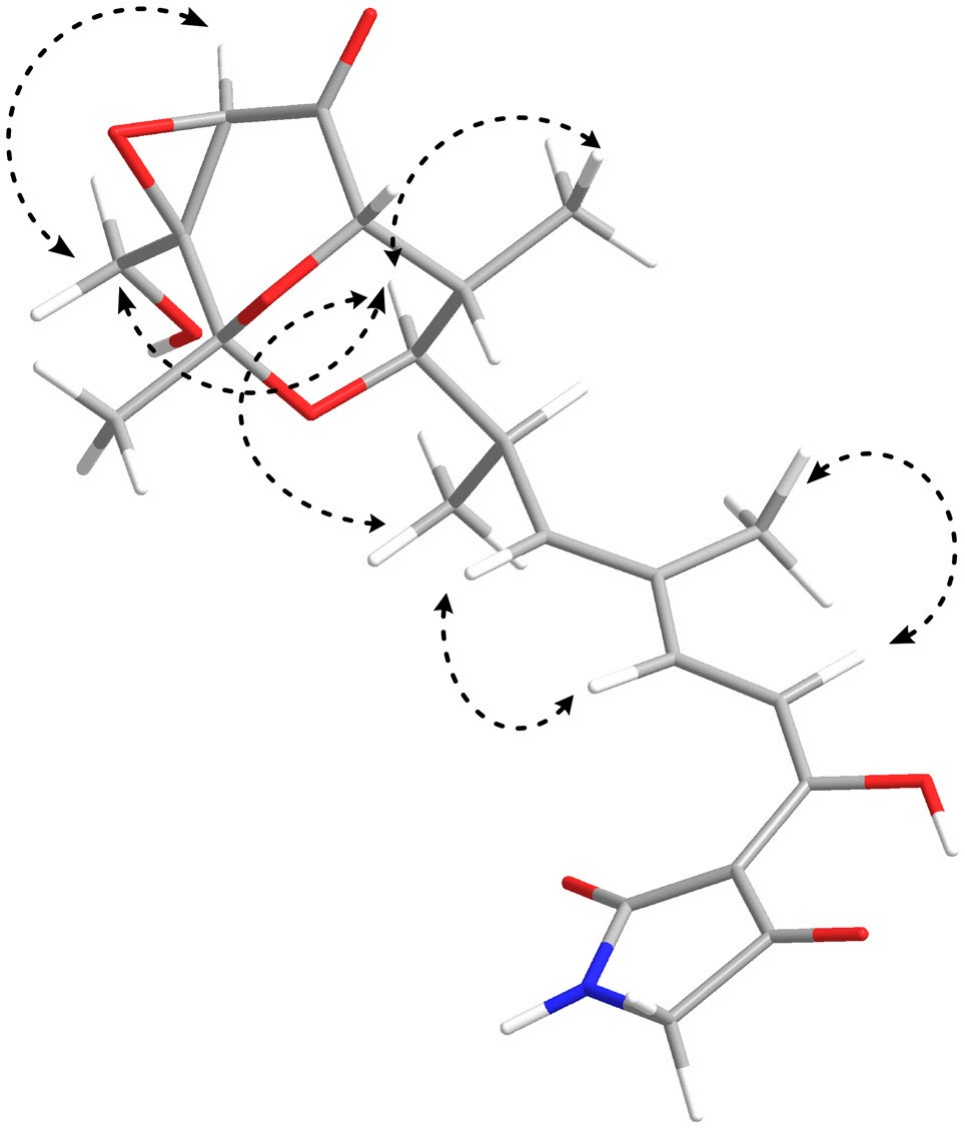
3D structure of compound **1** with the selected NOESY correlations.

**Figure 4:**
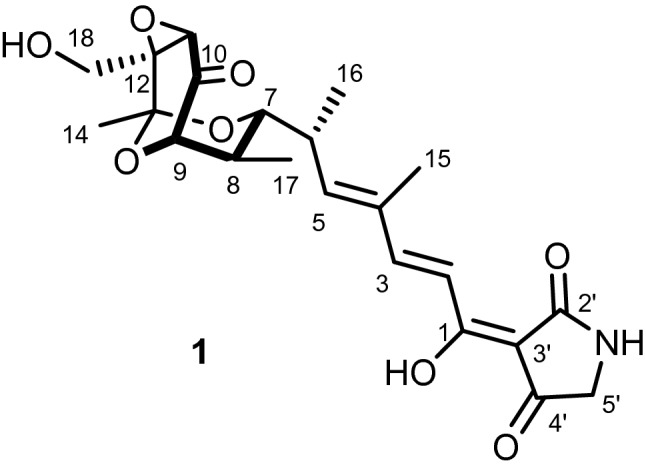
Chemical structures of compound **1** isolated from *Streptomyces* sp. SBST2-5^T^.

After purification of the crude extract using several chromatographic steps, compound **1** was isolated from the fermentation broth of *Streptomyces* sp. SBST2-5^T^. Compound **1** was identified based on 2D NMR spectroscopic analyses as well as mass spectrometry. The ^1^H and ^13^C NMR spectra (Additional files: Figs. S8 and S9) of compound **1** were identical to those of TAM B described in the literature^32^. The NOESY spectral information (Fig. 3) confirmed that compound **1** had the same relative configuration as that of the previously reported for TAM B^32^. HRESIMS spectrum (Additional file: Fig. S10) showed a negative ion at *m/z* 432.1662 [M−H]^−^, confirming the molecular formula C_22_H_27_NO_8_. Moreover, the optical rotation value of compound **1** ([α]_D_ − 30.19, MeOH; [α]_D_ − 14.64, EtOH) was the same as that of TAM B ([α]_D_ − 14, EtOH)^32^. Thus, compound **1** was identified as (−)-TAM B (Fig. 4). The complete ^1^H and ^13^C NMR spectral data of compound **1** were provided in additional file: Table S7. Many TAM analogs have been found in various strains of *Streptomyces* spp. such as *S. flaveolus*^33^, *Streptomyces* sp. 307–9^15^, *Streptomyces* sp. SCSIO1666^34^, *Streptomyces* sp. SCSIO 41399^17^, and *S. tirandamycinicus*^35^. It was also suggested that TAM is biosynthetically generated from hybrid polyketide synthase (PKS)/ non-ribosomal peptide synthase (NRPS)^34^. Furthermore, several previous reports indicated that TAM B had potent antibacterial activity against several strains of bacteria^17,32,35,36^, but there are no reports on its neuroprotective and anti-BACE1 activities.

### Neuroprotective, anti-acetylcholinesterase (anti-AchE), anti-β-secretase (anti-BACE1), antioxidative, and cytotoxic activities of TAM B

Alzheimer’s disease (AD) is one of the major health problems related to age cognitive dysfunction, which involves the decrease of acetylcholine (ACh) neurotransmitters, the accumulation of beta-amyloid (Aβ), and neurofibrillary triangle, and the occurrence of oxidative stress^37^. A promising way to treat these problems is to protect neurons in the brain. Actinomycetes have been reported as valuable sources of neuroprotective agents^6–9^. Recently, *Microbispora hainanensis* strain CSR-4 has been reported to be an outstanding actinomycete that produces a new diterpene compound, 2α-hydroxy-8(14), 15-pimaradien-17, 18-dioic acid. This compound displayed acetylcholinesterase (AChE) inhibitory activity with IC_50_ values of 96.87 ± 2.31 μg/mL^38^. *Microbispora* sp. TBRC 6027 is a potential actinomycete that produces several new chromone derivatives revealing neuroprotective activity. These compounds can protect P19-derived neurons from the oxidative stress induced by serum deprivation at very low concentrations (1 ng/mL)^39^. As part of our continuing work on the search for neuroprotective compound(s) from the actinomycetes, the EtOAc crude extract of *Streptomyces* sp. SBST2-5^T^ displayed neuroprotective ability against oxidative stress (OS) conditions, with %viability of 109.83 ± 1.24% at 1 ng/mL, implying the presence of compound(s) with the ability to prevent neuronal cell death and led us to isolate the major component, TAM B. TAM B was tested for its effect on the viability of the P19-derived neuron at various concentrations (1–10,000 ng/mL). The result showed that TAM B at all the tested concentrations exhibited no neurotoxicity on the cultured neuron. TAM B at various concentrations of 1–10,000 ng/mL expressed neuron viability range from 95.42 ± 13.74% to 116.53 ± 11.60%. Moreover, at a concentration of 1 ng/mL, TAM B promoted neuron viability higher than that of the control (TAM B showed 116.53 ± 11.60% neuron viability) (Additional file: Fig. S11). Thus, TAM B at 1 ng/mL was further evaluated for its neuroprotective ability by serum deprivation and Aβ_1-42_ administration models. Removal of the serum from the cultured neuron condition can cause neurotoxicity by inducing oxidative stress to the cultured P19-derived neuron^2,40,41^. In addition, Aβ also induced oxidative damage to the neuron^42^. TAM B at 1 ng/mL significantly (p < 0.05 compared with oxidative stress condition) helped to protect the cultured P19-derived neuron from the oxidative stress induced by serum deprivation. 1 nM Quercetin was used as a positive control, which possessed 77.01 ± 14.74% neuron viability, while the neuron cultured in the medium without serum (an oxidative stress condition) showed 36.30 ± 4.97% neuron viability. TAM B at 1 ng/mL exhibited neuron viability at 102.10 ± 9.83% (Additional file: Fig. S12). The neuroprotective ability against Aβ_1–42_ of TAM B was also evaluated. Aβ_1–42_ at 4.5 µM (4500 nM) showed toxicity on cultured P19-derived neurons by revealing neuron viability at 74.74 ± 9.05%. Co-administration of 4.5 µM Aβ_1–42_ with 1 ng/mL of TAM B showed great neuroprotective ability against Aβ_1–42_ by exhibiting a neuron viability of 112.72 ± 6.83% (Additional file: Fig. S13). The in vitro anti-acetylcholinesterase (anti-AchE), anti-β-secretase (anti-BACE1), and anti-oxidant (DPPH radical scavenging assay) activities of TAM B were evaluated for preliminary studying of the mechanism of action of the compounds. In our experiment, it was found that TAM B (250 μg/mL) had very low AChE inhibitory activity (5.36 ± 1.29%), while the positive control, galantamine at 1 µg/mL, showed AChE inhibitory activity of 75.98 ± 1.65%. Likewise, TAM B displayed low anti-oxidation activity with an IC_50_ of 166.30 µg/mL. These values suggested that TAM B was not responsible for anti-AChE and DPPH radical scavenging ability because no anti-AChE and anti-oxidation activities were detected. Interestingly, TAM B (20 µg/mL) exhibited BACE1 inhibitory activity of 68.69 ± 8.84%. In comparison, quercetin (positive control) at 20 µg/mL showed a value of 67.56 ± 9.40% for BACE1 inhibitory activity, indicating that TAM B had potential anti-BACE1 activity (Additional file: Table S8). In vitro cytotoxicity assays are used to predict the toxicity and assess compounds' safety to various host cells^43^. It is known that a valuable natural product should have no effect on cellular metabolism and exhibit no toxicity against the host. The effect of TAM B on cell viability was evaluated using the Vero cell line and the human embryonic kidney cell line, HEK293. TAM B at 1000 μg/mL displayed very low cytotoxic activity against Vero cells (%cell viability > 85%). In the case of cytotoxicity on HEK293 of TAM B, we found that TAM B at various concentrations of 0–10 μg/mL exhibited HEK293 cell viability range from 64.84 ± 12.64% to 99.88 ± 7.49% (Additional file: Fig. S14). In addition, Yu et al.^36^ have reported that TAM B shows no cytotoxicity against human hepatic cells. This evidence emphasizes that TAM B is a valuable microbial natural product and shows significant potential to act as a therapeutic drug for AD treatment. According to the anti-oxidation, anti-AchE, anti-BACE1, and neuroprotective activities of TAM B, we suggested that TAM B had a potential for AD treatment through neuroprotective and anti-BACE1 activities.

### Molecular docking and in silico assessment of binding mode between TAM B and human BACE1

**Figure 5:**
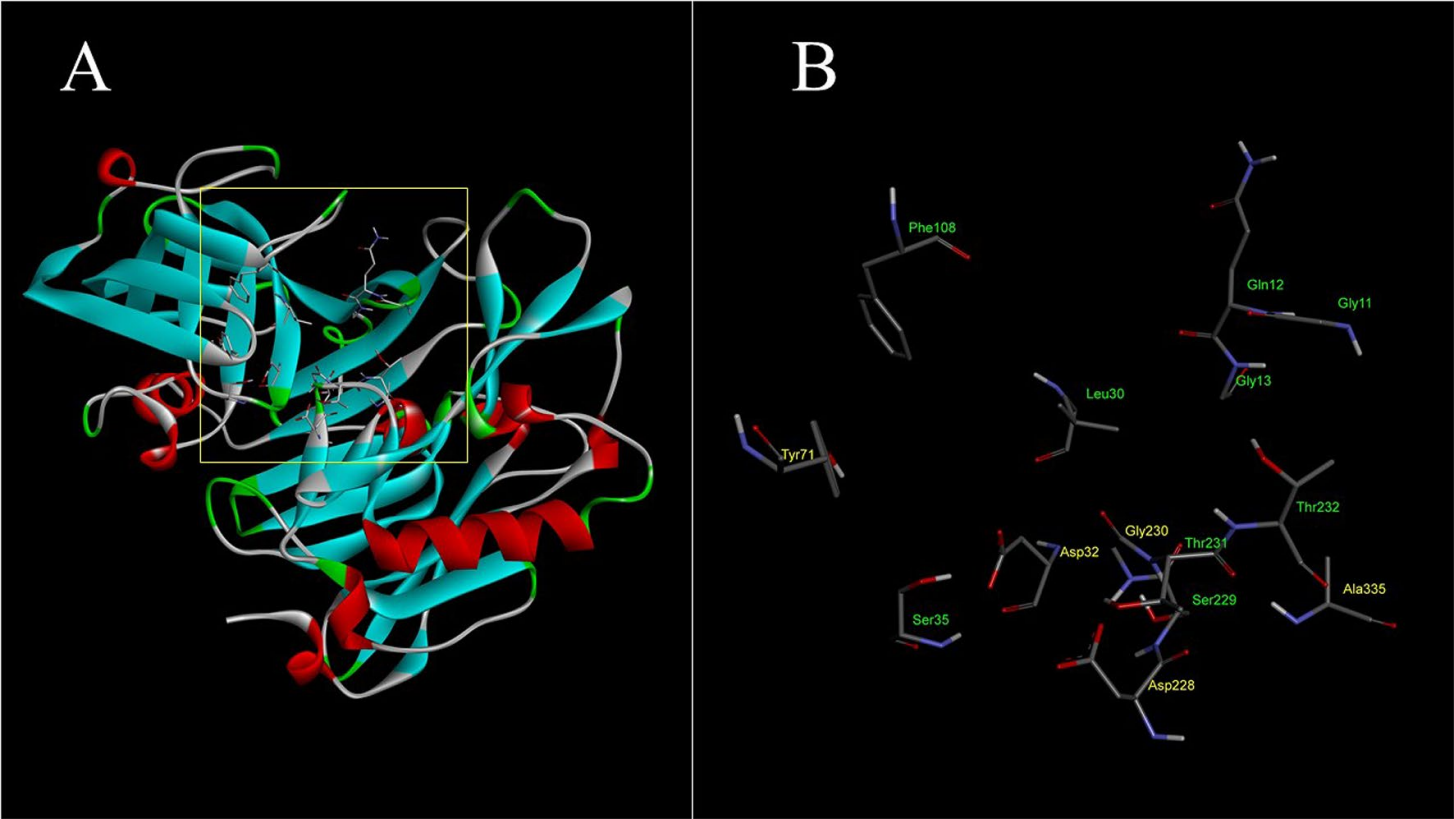
The three-dimensional structure of human BACE1 representing the search space size of 15 × 15 × 15 Å covering the binding site area for docking protocol (**A**). The amino acid residues lining in the binding site were depicted, including key residues (Asp32, Tyr71, Asp228, Gly230 and Ala335) for binding of atabecestat (**B**).

**Figure 6:**
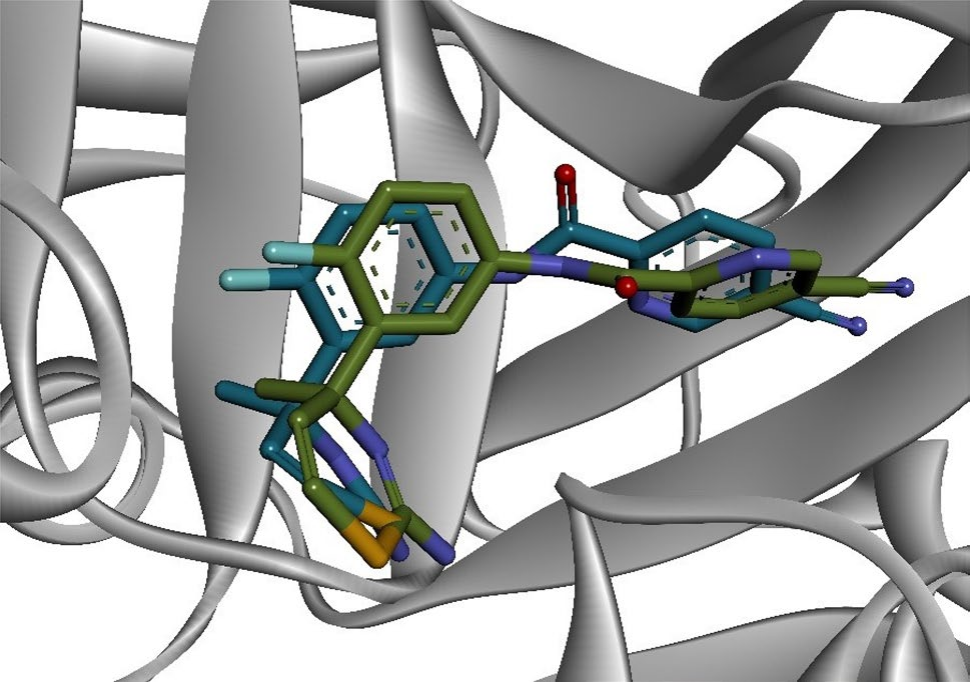
The reliability of docking protocol represented by the re-docked pose of atabecestat into the binding site of human BACE1. The structures of atabecestat were represented in stick by superposition of docking pose (green-carbon) and co-crystallized structure (cyan-carbon).

**Figure 7:**
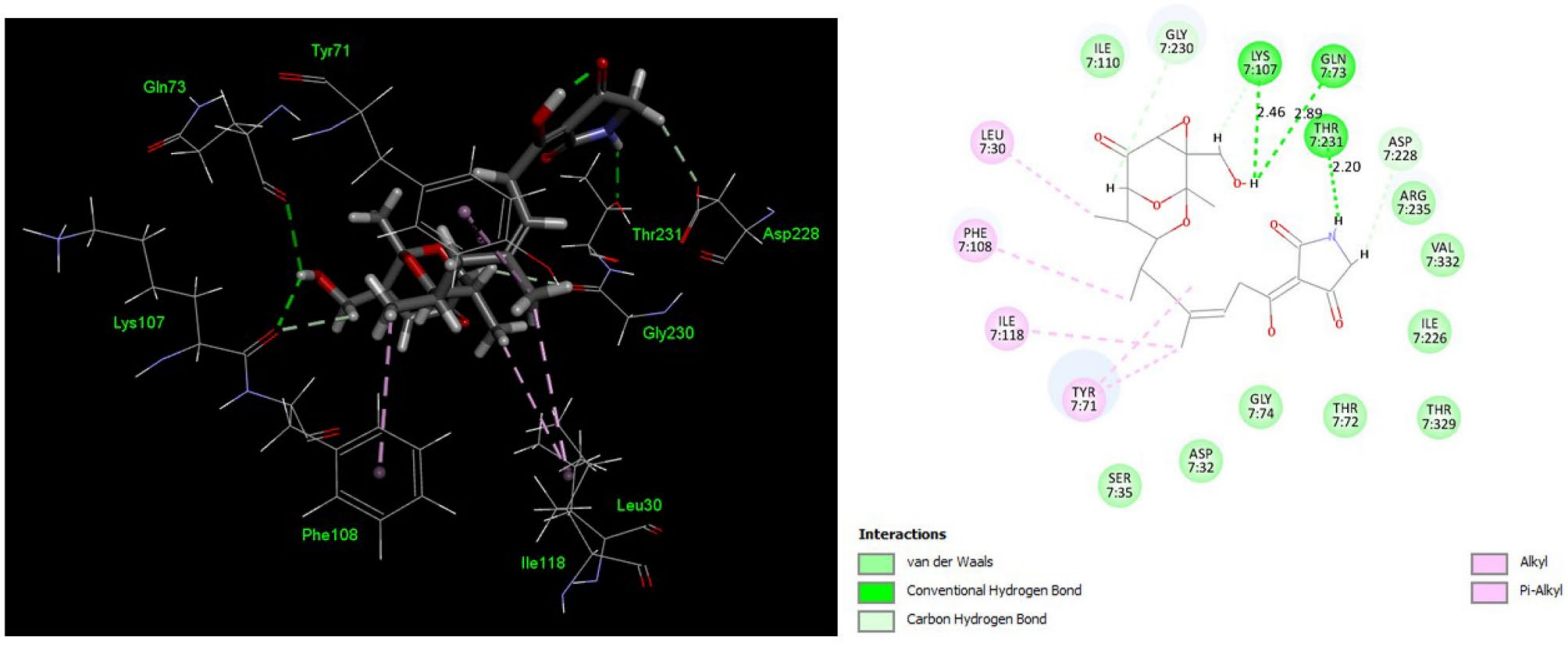
The binding mode of TAM B in the binding site of human BACE1. The binding residues within a radius of 4 Å from the bound TAM were illustrated in 3D graphic (**A**) and 2D diagram (**B**), indicating the types of binding interactions and interacting amino acids in the active site of human BACE1.

Based on the experimental part, TAM B exhibited a potential effect as an inhibitor for human BACE1. Molecular modeling was applied for a virtual understanding of the binding mode and interactions of TAM B in the binding site of human BACE1 using molecular docking and receptor-ligand interactions analyses. To assess the reliability of the docking protocol used in this study, a three-dimensional structure of energy-minimized atabecestat (PubChem CID 68254185) was docked into the binding site of prepared and energy minimized human BACE1 in apo form. The center coordinate and size of search space for docking were generated by covering the active sites of human BACE1 as represented in Fig. 5. The amino acid residues lining in the binding site were shown, including Asp228, Asp32, Tyr71, Gly230, and Ala335, which were identified as key residues for binding of atabecestat, a recently efficacious BACE1 inhibitor that was entered into the EARLY Phase 2b/3 clinical trial for the treatment of preclinical AD patients^44^. Among 20 docking poses, the best-docked pose of atabecestat presented the lowest negative binding energy score of − 8.0 kcal/mol (Additional files: Table S9 and Fig. S15). The docked conformation with respect to the X-ray crystal conformation of atabecestat in the binding site of human BASE1 (PDB ID: 7CDZ) showed the heavy atom RMSD value of 1.43 Å (Fig. 6) with well-oriented alignment indicating a significantly reliable protocol since the threshold of reliability was 2.0 Å for a good docking^45^. The structure of energy-minimized TAM B (PubChem CID 54728535) was then docked into the binding site of human BACE1 using the same protocol as atabecestat docking condition. The results of docking analyses showed that all binding energies of 20 docked poses of TAM B were in the range of − 6.8 to − 8.3 kcal/mol (Additional files: Table S9 and Fig. S16). The best docking pose of TAM B in the binding site of human BACE1, showing the binding energy score of − 8.3 kcal/mol was selected. To better understand the binding mode and interaction of TAM B, the predicted structure of tirandamycin-human BACE1 complex was submitted for further energy minimization prior to the analysis of binding interactions within 3.5 Å around TAM B. The interactions of the docked TAM B inside the binding site of BACE1 are shown in Fig. 7. The results showed that the NH_2_ group on the pyrrolidine-2,4-dione ring of the ligand showed a conventional hydrogen bond with the OH group of Thr231 at distances of 2.20 Å. This NH_2_ group was also in the vicinity of Asp 228, which formed a carbon-hydrogen bond with the hydrogen in the ring. Hydrogen bonds were observed between the hydroxy methyl group of TAM B and the CO group of Gln73 and Lys107 at distances of 2.89 and 2.46 Å, respectively. Leu30, Tyr71, Phe108, and Ile 118 mediated the alkyl and mixed pi-alkyl hydrophobic interactions. The calculated binding energy (BE) between TAM B and human BACE1 was − 105.66 kcal/mol. Moreover, the interaction energy between sets of atoms across all conformations was calculated using CHARMm. The interaction energy is defined as the sum of the van der Waals (VDW) and electrostatic energy. This nonbonded interaction energy was reported as energy values for each amino acid residue in the binding pocket within 3.5 Å interacting with TAM B (Table 1). The total interaction energy of − 62.82 kcal/mol was found. It comprised total VDW interaction energy and total electrostatic interaction energy of − 25.58 and − 37.24 kcal/mol, respectively. Considering molecular docking and binding mode analysis of tirandamycinB in human BACE1, all results indicated that TAM B could be a potential active human BACE1 inhibitor.

**Table 1:** Interaction energies per-residue within 4 Å of TAM B in the binding site of human BACE1.

Residue	Total interaction energy (kcal/mol)	VDW interaction energy (kcal/mol)	Electrostatic interaction energy (kcal/mol)
LEU30	− 1.44	− 0.85	− 0.59
ASP32	− 7.71	− 1.10	− 6.61
SER35	− 2.04	− 0.56	− 1.48
TYR71	− 6.57	− 4.55	− 2.02
GLN73	− 6.14	− 1.40	− 4.73
GLY74	− 0.53	− 0.43	− 0.10
LYS107	− 5.66	− 1.88	− 3.78
PHE108	− 1.34	− 2.52	1.18
ILE110	− 2.15	− 1.45	− 0.70
ILE118	− 2.27	− 1.14	− 1.13
ILE226	− 1.19	− 0.80	− 0.39
ASP228	− 13.41	− 1.39	− 12.03
GLY230	− 1.07	− 1.55	0.48
THR231	− 7.21	− 2.56	− 4.65
ARG235	− 2.77	− 1.85	− 0.92
VAL332	− 1.32	− 1.54	0.23
SUM	− 62.82	− 25.58	− 37.24

### Preliminary in silico pharmacokinetic ADME of TAM B

The preliminary pharmacokinetics ADMET properties of TAM B were predicted using the ADMETlab 2.0 web-based application^46^. The predicted physicochemical characteristics and ADMET properties were included (Additional file: Fig. S13). Based on Lipinski`s Rule for the central nervous system drugs (RoCNS), TAM B revealed closely CNS drug-likeness properties i.e., molecular weight = 433.170 (MW < 400), LogP = 3.081 (CLogP ≤ 5), a number of H-bond acceptor = 9 (HBA ≤ 7) and a number of H-bond donor = 4 (HBD ≤ 5)^47,48^. However, the topological polar surface area (TPSA) of compound 1 was 141.610 Å^2^ which was higher than 90 Å^2^ as a cut-off for optimal CNS exposure^49^. TAM B contains a number of rotatable bonds of 6 that was well agreed with the proposed guideline of a rotatable bond count < 8 as an attribute of a successful CNS drug candidate^50^. The distribution of TAM B was predicted as the probability of being blood–brain barrier (BBB) penetration. Accordingly, TAM B may be further optimized and/or experimentally measured in the BBB permeability to develop as a new lead for CNS active human BACE1 inhibitor in the field of Alzheimer’s disease pharmacotherapy.

### Description of *Streptomyces composti* sp. nov

*Streptomyces composti* (com.pos’ti. N.L. gen. n. *composti* of compost).

Cells are Gram-stain-positive and aerobic. It grows well on ISP 2, ISP 4, and nutrient agar. Grows moderately on ISP 3, ISP 5, ISP 6, and ISP 7. The growth on Czapek’s sucrose and glucose-asparagine agar is poor. Yellowish-grey series substrate mycelium is observed on ISP 2, ISP 3, ISP 5, ISP 6, and ISP 7. Grayish white to light grey aerial spore masses are formed on ISP 2, ISP 4, ISP 6, ISP 7, and nutrient agar that differentiate into rectiflexibiles spore chains with a hairy surface, and spores are non-motile. The light brown diffusible pigment is observed on ISP 2. The reduction of nitrate, and production of catalase are positive. Negative results are observed for oxidase activity, liquefaction of gelatin, hydrolysis of starch, peptonization of milk, and urease production. Decomposes adenine, hypoxanthine, and l-tyrosine but not cellulose and xanthine. Utilizes l-arabinose, d-galactose, d-glucose, *myo*-inositol, inulin, d-mannitol, d-mannose, sucrose, d-trehalose, xylitol, and d-xylose; weakly utilizes adonitol, d-cellobiose, d-lactose, d-melezitose, d-melibiose, d-raffinose, d-salicin; but does not utilize dextran, l-rhamnose, d-ribose as sole carbon sources. Utilizes l-arginine, l-asparagine, l-histidine, l-methionine, l-phenylalanine, l-proline, l-serine, l-threonine, and l-valine; weakly utilizes DL-2-aminobutyric acid; but does not utilize l-cysteine and 4-hydroxyproline as sole nitrogen sources. The growth temperature is between 20 and 55 °C. Maximum NaCl for growth is 6% (w/v). The pH range for growth is 5–10. According to the API ZYM system, cells show acid phosphatase, alkaline phosphatase, β-galactosidase, α-glucosidase, and leucine arylamidase activities. Cells produce weakly cystine arylamidase, esterase (C4), *N*-acetyl-β-glucosaminidase, and valine arylamidase but no activities of cells on α-chymotrypsin, esterase lipase (C8), α-fucosidase, α-galactosidase, β-glucosidase, β-glucuronidase, lipase (C14), α-mannosidase, naphthol-AS-BI-phosphohydrolase, and trypsin are observed. Cell wall peptidoglycan contains *LL*-diaminopimelic acid. The major menaquinones are MK-9(H_6_) and MK-9(H_8_), while MK-9(H_4_) is minor component. Galactose, glucose, mannose, and ribose are detected as whole-cell sugars. The phospholipid profile contains diphosphatidylglycerol, phosphatidylethanolamine, phosphatidylglycerol, phosphatidylinositol, phosphatidylinositol mannoside, and three unidentified phospholipids. The major fatty acids (> 10%) are *iso*-C_15:0_, *iso*-C_16:0_, *anteiso*-C_15:0_. The DNA G+C content of the type strain is 72.2%.

The type strain, SBST2-5^T^ (= TBRC 9952^T^ = NBRC 113999^T^), is an actinomycete isolated from the wastewater treatment bio–sludge compost collected from Suphanburi province, Thailand. The GenBank accession number for the 16S rRNA gene sequence of strain SBST2-5^T^ is LC430996. The whole-genome shotgun project has been deposited at GenBank under the accession JAATEM000000000.

## Conclusions

In conclusion, we herein reported the identification and genome properties of *Streptomyces* sp. SBST2-5^T^. The genome-based taxonomic characterization revealed that *Streptomyces* sp. SBST2-5^T^ merits classification as a novel species of the genus *Streptomyces*, for which we propose the name *Streptomyces composti* sp. nov. The strain represents a hybrid PKS/NRPS gene cluster in its genome involved in the TAM production, and led to the isolation of TAM B as a significant component in its EtOAc extract. To our knowledge, this is the first report of TAM B neuroprotective properties, reducing the toxicity of Aβ_1–42_, protecting the neuron from oxidative stress induced by serum deprivation and possessing anti-BACE1 activity, which position the compound to be used as multi-target anti-AD agents. An in silico study based on molecular docking simulation was performed to predict the binding mode of the compound into the binding pocket of the BACE1 enzyme and revealed that some amino acids of the BACE1 interact with the compound. Therefore, It is concluded that TAM B could be proposed as a potential active BACE1 inhibitor.

## Experimental procedures

### Isolation, cultivation, and preservation of ***Streptomyces*** sp. SBST2-5^T^

Strain SBST2-5^T^ was isolated from the wastewater treatment bio–sludge compost collected from Suphanburi province, Thailand (14° 38′ 06ʺ N and 99° 50′ 45ʺ E). The air-dried compost was heated at 100 °C for 1 h. The diluted 1000-fold compost solution was prepared by serial dilution technique with 0.01% sterile sodium dodecyl sulfate (SDS) in distilled water, and an aliquot (0.1 mL) of the sample suspension was taken and spread onto Zhang’s starch soil extract (ZSSE) medium^51^ supplemented with 50 mg nalidixic acid and 50 mg/L nystatin. After incubation at 30 °C for 14 days, a small light grey colony of strain SBST2–5^T^ was picked and purified on yeast extract–malt extract agar (International *Streptomyces* Project, ISP 2 medium)^52^. The purified isolate was preserved at 4 °C on ISP 2 slant and in glycerol 20% (v/v) suspension at – 80 °C or freeze-drying for long-term preservation. *Streptomyces* sp. SBST2-5^T^ is deposited in Thailand Bioresource Research Center (TBRC) and NITE Biological Resource Center (NBRC) for code numbers TBRC 9952 and NBRC 113999, respectively (Related files: Figs. R1 and R2).

### Polyphasic taxonomic characterizations of strain SBST2-5^T^

#### Morphological, cultural, physiological, and biochemical characteristics

Spore morphology was observed by a scanning electron microscope (SEM) using the culture grown at 30 °C for 14 days on ISP 2 agar. Samples for scanning electron microscopy were prepared according to the method of Duangupama et al*.*^53^. The arrangement of spore was observed under a scanning electron microscope (model JSM-6610 LV; JEOL). The cultural characteristic was determined by cultivating strain SBST2-5^T^ on various International *Streptomyces* Project (ISP) media (ISP 2–7)^52^ at 30 °C for 14 days. The ISCC-NBS color charts were used for determining color designations and names^54^. Growth at different temperatures (4–60 °C) and different concentrations of NaCl [0–10% (w/v) at increments of 1%] were evaluated on ISP 2 agar after incubation for 14 days. Growth at different pH (4.0–11.0 at an increment of 0.5 pH unit) was determined by cultivation at 30 °C for 14 days in ISP 2 broth using acetate buffer (pH 4.0–5.0), phosphate buffer (pH 6.0–7.0, Tris–HCl (pH 8.0–9.0), glycine–NaOH buffer (pH 10.0) and carbonate bicarbonate buffer (pH 11.0) instead of distilled water. Carbon and nitrogen utilization (1%, w/v), nitrate reduction, gelatin liquefaction, hydrogen sulfide and urease production, the decomposition of adenine, cellulose, hypoxanthine, L-tyrosine, and xanthine were evaluated using the previously described methods^55–57^. The reference strain, *S. thermoviolaceus* NBRC 13905^T^, was cultured under the same conditions for comparative analyses.

#### Chemotaxonomic and 16S rRNA gene analyses

Dried cells of strain SBST2-5^T^ and *S. thermoviolaceus* NBRC 13905^T^, the reference strain, were prepared by cultivation in ISP2 broth on a rotary shaker (200 rpm) at 30 °C for 5 days. The isomer of diaminopimelic acid (DAP) in the peptidoglycan was evaluated using the methods of Hasegawa et al.^58^. Whole-cell sugar analysis was performed using the TLC method suggested by Komagata and Suzuki^59^. The polar lipids were extracted and analyzed using the standard protocols of Minnikin et al.^60^ and Collins and Jones^61^. To extract the menaquinone in the cells, the method of Collins et al.^62^ were used. The type of menaquinone was analyzed by high-performance liquid chromatography (HPLC)^63^. To prepare the cells for fatty acid analysis, strain SBST2-5^T^ and its related type strain were cultivated in ISP 2 broth on a rotary shaker (200 r.p.m.) at 30 °C for five days. The Sherlock Microbial Identification (MIDI system and the ACTIN version 6 database were used for determining the fatty acid components^64,65^. Genomic DNA was isolated from cells grown in ISP 2 broth according to the method of Tamaoka^66^. The amplification and sequencing of the 16S rRNA gene were carried out using the method suggested by Nakajima et al.^67^. For calculating and comparing levels of similarity, the 16S rRNA gene sequence of strain SBST2-5^T^ was analyzed using the EzBioCloud server^68^. The neighbour-joining (NJ), and maximum-likelihood (ML) trees based on 16S rRNA gene sequences were aligned and reconstructed using MEGA X^69^. The evolutionary distances among the strains were analyzed using Kimura’ s two-parameter method^70^. The confidence values of the branches were determined using 1000 replications of the bootstrap resampling method^71^.

#### Genome-based taxonomic characterization and genome analysis for secondary metabolites of strain SBST2-5^T^

Genomic DNA of strain SBST2-5^T^ was sequenced using an Illumina Miseq platform (Illumina, Inc., San Diego, US-CA) with Reagent Kit V3 (600 cycles) using 2 × 250 bp paired-end reads (Chulalongkorn University, Thailand). Sequencing libraries were prepared using the QIAseq FX DNA Library Kit (Qiagen, USA). 100 ng of gDNA was subjected to DNA sequencing library preparation using QIASEQ FX DNA library preparation kit (Qiagen, USA). The quality of the raw reads was checked using FASTQC (Babraham Bioinformatics). Trim Galore (Babraham Bioinformatics) was used to remove the low-quality read and adaptors. SPAdes^72^ was used for assembling the genome. Genome annotation reports were created by the Prokka software 1.12^73^. The average nucleotide identity (ANI) result was calculated using the Jspecies^74^. The average amino acid identity (AAI) value was evaluated using the Kostas Lab ANI calculator^75^. The digital DNA-DNA hybridization (dDDH) values were calculated using the genome-to-genome distance calculator (GGDC 2.1; blast + method)^76^ on the TYGS type strain genome server (https://tygs.dsmz.de/)^77^. The presence of biosynthetic gene clusters in the genome of strain SBST2-5^T^ was analyzed using antiSMASH^30^. To determine the genes related to tirandamycin production, the genome of strain SBST2-5^T^ was analysed using blastp on the Uniprot database with matrix; blosum62 (https://www.uniprot.org/blast)^78^. To analyze the taxonomic position of strain SBST2-5^T^ in phylogenomic tree, the genomes affiliated with the genus *Streptomyces* were downloaded from NCBI, and the genome quality was checked using CheckM v1.0.1^79^. Genomes of 10% contamination were discarded from the study. To construct the phylogenomic tree, an automated multi-locus species tree (autoMLST) pipeline (https://automlst.ziemertlab.com/)^80^ was used. The final phylogenomic tree was reconstructed using the maximum-likelihood algorithm using MEGA X, with *Micromonospora carbonacea* DSM 43168^T^ (GCF_900091535) as an outgroup.

#### Fermentation, extraction, and isolation of a bioactive substance

*Streptomyces* sp. SBST2-5^T^ was grown on ISP2 agar for 7 days. The agar was cut into small pieces and then transferred into 10 × 1 L Erlenmeyer flasks, each containing 250 mL of ISP 2 broth. The seed culture was cultivated at 30 °C on a rotary shaker at 180 rpm for 6 days. Then 25 mL of seed culture was transferred into 80 × 1 L Erlenmeyer flasks, each containing 250 mL of ISP2 medium. The flaks were shaked at 30 °C, 200 rpm, and after 6 days, the whole culture was extracted three times with an equal volume of EtOAc, which was later dried over Na_2_SO_4_ and evaporated to yield a reddish-brown gum (1.74 g). The gum was passed through a Sephadex LH20 column (1.5 × 40 cm), eluted with 100% MeOH, and 10 mL fractions were collected for 100 tubes. Each tube was analyzed by HPLC and then concatenated to give two fractions. Both fractions were further purified by a preparative HPLC, using a Sunfire C18 column (diam. 19 × 250 mm, particle size 10 mm) and eluted with a linear gradient system of 5–70% CH_3_CN in water over 21 min to furnish compound **1** (*t*_R_ 19.38 min, 163.3 mg).

*(−)-Tirandamycin B (1)*: pale yellow solid; [α]^25^_D_ − 30.19 (*c* 0.34, MeOH) and [α]^27^_D_ − 14.64 (*c* 0.16, EtOH); UV (MeOH) λmax (log ε) 202 (4.03), 250 (3.75), 290 (3.82), 339 (4.10); HRESIMS *m/z* 432.1662 [M − H]^−^ (calcd for C_22_H_26_NO_8_, 432.1664).

#### Structure identification of the active compound

UV spectrum was performed in MeOH on a Spekol 1200 spectrophotometer from Analytik Jena. Optical rotation was measured with a JASCO P-1030 digital polarimeter. FT-IR spectrum was measured on a Bruker ALPHA spectrometer. NMR spectra were acquired in CDCl_3_ on a Bruker Avance 500 MHz NMR spectrometer. HRESIMS data was determined on a Bruker MicrOTOF mass spectrometer.

### In Vitro determination of biological activities

#### Antioxidant assay

The antioxidant capacity was estimated in terms of radical scavenging activity according to a modified version of Brand-Williams method^81^. Briefly, 100 µL of tested compound (1–1000 µg/mL dissolved in MeOH) was mixed with 100 µL of freshly prepared DPPH solution (3 × 10^−5^ M dissolved in MeOH). The reaction mixture was incubated for 30 min. The absorbance was read at 517 nm. Each assay was done in triplicate. Ascorbic acid was used as a positive control.

#### Anti-acetylcholinesterase (anti-AChE) assay

The AChE inhibitory effect was modified from the spectrophotometric assays described by Panyathip et al*.*^2^. In brief, 25 µL of 0.2 U/mL AChE from *Electrophorus electricus* and 25 µL of sample dissolved in 50 mM TRIS–HCl buffer pH 8.0 were added to the 96-well and incubated at 37 °C for 15 min. Then 125 µL of 3 mM 5,5′-dithiobis-(2-nitrobenzoic acid) (DTNB) and 25 µL of 1.5 mM acetylthiocholine iodide (ATCI) were added into the 96-wells and incubated at 37 °C for 15 min. The absorbance was measured at 405 nm by a microplate reader (Spectramax, USA). Galanthamine (8 µg/mL in 50 mM TRIS–HCl buffer pH 8.0) was used as the positive control. Each assay was done in 3 independent experiments, and each experiment was run in triplicate. Data were reported as average % inhibition ± SD.

#### Beta-secretase 1 (BACE1) inhibition assay

The BACE1 inhibitory effect was modified from the assays described by Puksasook et al.^1^ and Panyathip et al.^2^. In brief, 20 µL of 0.1 U/mL BACE1 and 20 µL of sample dissolved in 100 mM sodium acetate buffer pH 4.5 were added to the 96-well and incubated at 37 °C for 10 min. Then 50 µL of 750 mM β-secretase substrate in 100 mM sodium acetate buffer pH 4.5 was added and incubated at 37 °C for 20 min. After that, 10 µL of stop buffer (2.5 M sodium acetate buffer pH 4.5) was added to the 96-wells and measured the fluorescence at 380 nm (excitation wavelength) and 510 nm (emission wavelength) by a microplate reader (Spectramax, USA). Quercetin (2 µg/mL) was used as the positive control. Each assay was done in 3 independent experiments, and each experiment was run in triplicate. Data were reported as average % inhibition ± SD.

#### Neuroprotective assay by serum deprivation method

The assay was performed in a 96-well plate for 3 independent experiments and each experiment was run in triplicate. First, the sample was evaluated for its ability to enhance the viability of the cholinergic P19-derived neuron by XTT reduction assay as described previously^2,82,83^ at various concentrations (1–10,000 ng/mL). Then, the concentration that enhances the viability of the neuron more than the %neuron viability of the control (0.5% DMSO in the medium was used as a control representing no effect on the neuron viability) will be selected to further evaluate for neuroprotective activity by serum deprivation method. Quercetin at 1 nM concentration was used as a positive control for the neuroprotective assay^82^. Data were reported as average %neuron viability ± SD.

#### Neuroprotective assay by Aβ_1–42_ co-administration method

The assay was performed in a 96-well plate for three independent experiments, and each experiment was run in triplicate. The concentration of Aβ_1–42_ at 4.5 µM that gave 70% neuron viability was used as a neurotoxin, and the sample at the concentration that enhanced the viability of the neuron more than the %neuron viability of the control (0.5%DMSO in the medium was used as a control representing no effect on the neuron viability) will be selected to further evaluate for neuroprotective activity by co-administration with 4.5 µM Aβ_1–42_. Data were reported as average % neuron viability ± SD.

#### Cytotoxicity assay

The assay was done in a 96-well plate for three independent experiments, and each experiment was run in triplicate. Human embryonic kidney cell line, HEK293 (ATCC CRL-1573), cultured in the conditions recommended by ATCC (Eagle’s minimum essential medium (EMEM) supplemented with 10%v/v FBS and 1%v/v antibiotic–antimycotic solution). The sample was evaluated for its cytotoxicity on cultured HEK293 cells by XTT reduction assay^2^ at various concentrations (1–10,000 ng/mL). 0.5% DMSO in the medium was used as a control representing no cytotoxic effect on the cultured cell. Data were reported as average % cell viability ± SD. The green fluorescent protein microplate assay^84^ (GFPMA) was used for testing cytotoxicity against Vero cells (African green monkey kidney fibroblasts, ATCC CCL-81). Ellipticine was used as a positive control for cytotoxicity against Vero cells. Ellipticine was used as a positive control for cytotoxicity against Vero cells. All experiments were done in triplicate, and data are presented as mean ± standard deviation (mean ± SD).

#### Structure preparations, molecular docking and binding mode analyses

The X-ray crystal structure of human BACE1 complexed with atabecestat (N-{3-[(4S)-2-amino-4-methyl-4H-1,3-thiazin-4-yl]-4-fluorophenyl}-5-cyanopyridine-2-carboxamide) (PDB ID: 7DCZ) was retrieved from the RCSB Protein Databank (PDB, https://www.rcsb.org/), and used as the target for docking of TAM B^44^. The three-dimensional structure of TAM B with desired configuration ((3E)-3-[(2E,4E,6R)-1-hydroxy-6-[(1S,2S,4R,6S,7R,8R)-2-(hydroxymethyl)-1,7-dimethyl-5-oxo-3,9,10-trioxatricyclo[4.3.1.02,4]decan-8-yl]-4-methylhepta-2,4-dienylidene]pyrrolidine-2,4-dione) was obtained from PubChem CID 54728535. The preparation of the human BACE1 structure was done using “Prepare Proteins” in BIOVIA Discovery Studio software package (BIOVIA)^85^. Briefly, the human BACE1 structure (PDB ID: 7DCZ) was cleaned up by removing hetero atoms, including water and atabecestat. The amino acids in missing loop regions of the human BACE1 chain (158-GFPLNQSEVL-167) and A272 were inserted based on the SEQRES data. The amino acid residues in human BACE1 were protonated at pH 7.4 according to the predicted pK values. The protein backbone atoms were restrained with a force constant of 40 kcal/mol. The force field and charges were assigned using CHARMm forcefield^86^ and Momany-Rone^87^, respectively. The prepared structure of human BACE1 was submitted for energy minimization with the conjugate gradient algorithms, 10,000 steps, and RMS gradient of 0.001 kcal/(mol × Å). The structure of TAM B was submitted for energy minimization using the conjugate gradient method with 3000 steps, RMS gradient 0.001 kcal/(mol × Å), applying CHARMm forcefield. The energy-minimized structures of a target (human BACE1) and ligand (TAM B) were saved. The plausible binding mode of TAM B in human BACE1 was predicted using the molecular docking technique. Docking of TAM B into the binding site of human BACE1 was performed using Autodock Vina^88^ interface in VEGA ZZ49^89^. The input files in pdbqt format for Autodock Vina were prepared using receptor.c and ligand.c scripts in VEGA ZZ platform for protein and ligand, respectively. The center of search space was setup at -40.19, -1.54 and 10.61 for X, Y, Z coordinates based on the binding mode of atabecestat in human BACE1 structure (PDB ID: 7DCZ)^48^. The search space was set up using 15 × 15 × 15 Å size around the center. Exhaustiveness and binding modes were set at 128 and 20 for ligand docking to generate ten output poses for ligand. The best-docked pose was selected based on energy scoring functions (kcal/mol) and protein–ligand interactions for further analyses. The structure of human BACE1-TAM B complex was submitted for energy minimization with CHARMm forcefield using the conjugate gradient (50,000 steps) to obtain the stable structure with a convergence criterion of 0.001 kcal/(mol × Å) energy RMS Gradient. Harmonic restraint was applied on the protein backbone during the minimization steps with 40 kcal/mol/Å^−2^. The binding energy (BE in kcal/mol) of the complex was estimated between ligands and target using CHARMm-based energy methods^90^. The nonbonded interactions (i.e., van der Waals and electrostatic terms) between the TAM B and interacting amino acid residues within 3.5 Å around the ligand were calculated using the CHARMm-based Interaction Energy protocol. The molecular modeling tools for structure preparation, energy minimization, calculations of binding and interaction energies, graphical visualization, and analyses were performed using the BIOVIA Discovery Studio package software (BIOVIA).

### Preliminary in silico ADME prediction

The preliminary pharmacokinetic ADME properties of TAM B were predicted using the ADMET lab 2.0 web-based application^46^.

## Supplementary Information

Supplementary Information.

## Data Availability

The datasets generated and/or analysed during the current study are available on the NCBI website and with the following accession codes at the NCBI database: *Streptomyces* sp. SBST2-5^T^: LC430996, JAATEM000000000; *S. thermoviolaceus* NBRC 13905^T^: Z68096, JAATEL000000000; *S. thermoviolaceus* JCM 4843^T^: BMVZ00000000; *S. thermoviolaceus* subsp. *apingens* DSM 41392^T^: Z68095; *S. thermocarboxydovorans* DSM 44296^T^: U94489; *S. thermospinosisporus* AT10^T^: AF333113; *S. nodosus* ATCC 14899^T^: CP009313-CP009313; *S. nodosus* DSM 40109^T^: JACHMR000000000; *S. chilikensis* RC 1830^T^: JN050256, LWCC00000000; *S. fragilis* NRRL 2424^T^: AY999917; *S. fragilis* NBRC 12862^T^: BEVZ00000000; *S. glaucus* LMG 19902^T^: AJ781332; *S. achromogenes* subsp. *rubradiris* NBRC 14000^ T^: AB184561; *S. mexicanus* CH-M-1035^T^: AF441168; *S. violaceoruber* NBRC 12826^T^: AB184174; *S. tricolor* NBRC 15461^T^: AB184687; *S. tendae* ATCC 19812^T^: D63873; *S. tendae* JCM 4610^T^: BMUY00000000; *S. anthocyanicus* NBRC 14892^T^: AB184631; *S. rubrogriseus* LMG 20318^T^: AJ781373; *S. lienomycini* LMG 20091^T^: AJ781353; *S. violaceorubidus* LMG 20319^T^: AJ781374; *S. tritolerans* DAS 165^T^: DQ345779; *S. lomondensis* NBRC 15426^T^: AB184673; *S. griseoflavus* LMG 19344^T^: AJ781322; *S. griseoincarnatus* LMG 19316^T^: AJ781321; *S. labedae* NBRC 15864^T^: AB184704; *S. abyssomicinicus* CHI39^T^: LC495888; *S. lasiicapitis* 3H-HV17(2)^T^: KX777589; *S. aureoverticillatus* NRRL B-3326^T^: AY999774; *S. calvus* JCM 4326^T^: BMSU00000000; *S. calvus* T-3018^T^: VCNP00000000; *S. aureorectus* DSM 41692^T^: JACJHU000000000; *S. asterosporus* DSM 41452^T^: CP022310; *S. albaduncus* JCM 4715^T^: BMVH00000000; *S. albaduncus* CECT 3226^T^: JACHJE000000000; *S. griseoloalbus* JCM 4480^T^: BMUD00000000; *S. griseostramineus* JCM 4385^T^: BMTK00000000; *S. griseostramineus* CECT 3273^T^: JACHJI000000000; *S. griseomycini* JCM 4382^T^: BMTI00000000; *S. matensis* JCM 4277^T^: BMSN00000000; *S. griseicoloratus* TRM S81-3^T^: JACVQF000000000; *S. rubradiris* JCM 4955^T^: BNCB00000000; *Streptomyces* sp. 307–9: ADC79637-ADC79649; *Actinopolyspora mortivallis* DSM 44261^T^: KB913024.

## Abbreviations

ACh: Acetylcholine
AD: Alzheimer’s disease
APP: Amyloid precursor protein
ANI: Average nucleotide identity
AAI: Average amino*-*acid identity
dDDH: Digital DNA–DNA hybridization
BACE1: Beta-secretase 1
BBB: Blood–brain barrier
CHCl_3_: Chloroform
DAP: Diaminopimelic acid
DSM: German collection of microorganisms and cell cultures GmnH
EtOAc: Ethyl acetate
FT-IR: Fourier transformation infrared spectroscopy
GC: Gas chromatography
ISP: International *Streptomyces* project
GFPMA: Green fluorescent protein microplate assay
MeOH: Methanol
MS: Mass spectrometry
XTT: Sodium 3′-[1-(phenylaminocarbonyl)-3,4-tetrazolium]-bis (4-methoxy6-nitro) benzene sulfonic acid hydrate
NAPAA: Non-alpha poly-amino acids like e-polylysin
NMDA: *N*-Methyl-d-aspartate
NMR: Nuclear magnetic resonance
NRPS: Non-ribosomal peptide synthetase
RNAp: Ribonucleic acid polymerase
smBGCs: Secondary metabolites biosynthetic gene clusters
TAM: Tirandamycin
T1PKS: Type I polyketide synthase
T2PKS: Type II polyketide synthase
T3PKS: Type III polyketide synthase
TBRC: Thailand Bioresource Research Center
USFDA: US Food and Drug Administration
