# Supplementary material for: New insights into the neuroprotective and beta-secretase1 inhibitor profiles of tirandamycin B isolated from a newly found Streptomyces composti sp. nov
Source: Sci Rep. 2023 Mar 24;13:4825. doi: 10.1038/s41598-023-32043-3 (PMC10038987; doi:10.1038/s41598-023-32043-3)
Supplement: Supplementary file 1 — Supplementary Information. [file 41598_2023_32043_MOESM1_ESM.pdf]

Scientific Reports (Additional information)

New insights into the neuroprotective and beta-secretase1 inhibitor profiles of tirandamycin B isolated from a newly found *Streptomyces composti* sp. nov.

Thitikorn Duangupama[1], Jaturong Pratuangdejkul[2], Sumet Chongruchiroj[2], Pattama Pittayakhajonwut[3], Chakapong Intaraudom[3], Sarin Tadtong[4], Patcharawee Nunthanavanit[5], Weerasak Samee[5], Ya-Wen He[6], Somboon Tanasupawat[7], Chitti Thawai[1,8*]

Affiliation

[1]Department of Biology, School of Science, King Mongkut's Institute of Technology Ladkrabang, Bangkok 10520, Thailand

[2]Department of Microbiology, Faculty of Pharmacy, Mahidol University, Phayathai Bangkok 10400, Thailand.

[3]National Center for Genetic Engineering and Biotechnology (BIOTEC), National Science and Technology Development Agency (NSTDA), Thailand Science Park, Phaholyothin road, Klong Luang, Pathum Thani, 12120 Thailand

[4]Department of Pharmacognosy, Faculty of Pharmacy, Srinakharinwirot University, Nakhon nayok 26120, Thailand

[5]Department of Pharmaceutical Chemistry, Faculty of Pharmacy, Srinakharinwirot University, Nakhon nayok 26120, Thailand

[6]State Key Laboratory of Microbial Metabolism, School of Life Sciences & Biotechnology, Shanghai Jiao Tong University, Shanghai 200240, P R China

[7]Department of Biochemistry and Microbiology, Faculty of Pharmaceutical Sciences, Chulalongkorn University, Bangkok, 10330, Thailand

[8]Actinobacterial Research Unit, School of Science, King Mongkut's Institute of Technology Ladkrabang, Bangkok 10520, Thailand

***Correspondence:** chitti.th@kmitl.ac.th

Department of Biology, School of Science, King Mongkut's Institute of Technology Ladkrabang, Bangkok 10520, Thailand

Additional information

Table S1 Cellular fatty acid compositions (%) of strain SBST2-5^T and *Streptomyces thermoviolaceus* NBRC 13905^T.

Cultures were grown in ISP 2 broth on a rotary shaker at 30 °C for 5 days. All fatty acid results were determined in this study. -, not detected.

**Fatty acid**	**% fatty acid of representative strains**	
	**Strain SBST2-5^T**	***S. thermoviolaceus* NBRC 13905^T**
**Saturated fatty acid**		
2OH-$C_{11:0}$	-	0.2
$C_{12:0}$	0.7	0.5
$C_{13:0}$	0.1	-
$C_{14:0}$	1.1	0.2
2OH-$C_{15:0}$	0.4	0.1
$C_{16:0}$	5.1	1.0
3OH-$C_{16:0}$	0.2	-
$C_{17:0}$	0.4	0.2
2OH-$C_{17:0}$	0.2	0.1
3OH-$C_{17:0}$	0.1	-
$C_{18:0}$	0.2	0.1
**Unsaturated fatty acid**		
2OH-$C_{16:1}$	0.2	-
iso-H-$C_{16:1}$	0.2	1.2
$C_{17:1}$ ω8c	0.1	0.1
iso-$C_{17:1}$ ω5c	-	0.1
anteiso-$C_{17:1}$ ω9c	0.3	3.1
$C_{18:1}$ ω9c	0.2	0.2
$C_{20:1}$ ω7c	0.2	0.2
**Branched fatty acids**		
iso-$C_{11:0}$	0.1	-
anteiso-$C_{11:0}$	-	0.2
iso-$C_{12:0}$	0.5	-
iso-$C_{13:0}$	1.6	0.1
anteiso-$C_{13:0}$	0.6	0.3
iso-$C_{14:0}$	9.8	1.7
anteiso-$C_{14:0}$	0.3	0.1
3OH-i-$C_{14:0}$	0.2	-
iso-$C_{15:0}$	27.4	9.2
anteiso-$C_{15:0}$	15.7	24.9
3OH-*iso*-$C_{15:0}$	0.5	0.1
iso-$C_{16:0}$	23.0	21.5
anteiso-$C_{16:0}$	-	0.2
3OH-*iso*-$C_{16:0}$	0.8	-
iso-$C_{17:0}$	4.4	6.0
anteiso-$C_{17:0}$	3.6	25.1

3OH-*iso*-$C_{17:0}$	0.5	0.1
iso-$C_{18:0}$	0.2	2.1
**10-Methyl fatty acids**		
$C_{17:0}$ cyclo	0.1	0.2
**Summed feature 3**	0.2	0.1
**Summed feature 9**	0.4	0.6

*Summed features are fatty acids that cannot be resolved reliably from another fatty acid using the chromatographic conditions chosen. The MIDI system groups these fatty acids together as one feature with a single percentage of the total. Summed Feature 3 comprised $C_{16:1}$ $\omega 6c$ and/or $C_{16:1}$ $\omega 7c$. Summed Feature 9 comprised 10-methyl $C_{16:0}$ and/or iso-$C_{17:1}$ $\omega 9c$.

Table S2 ANIb, AAI and dDDH values of the *Streptomyces* sp. SBST2-5^T and related type strains.

Query genome	Reference genome	ANIb (%)	AAI (%)	Digital DNA-DNA hybridization relatedness Formula 2				G+C difference
				% dDDH	Model C.I. (%)	Distance	Prob. DDH ≥ 70 (same species)	
Streptomyces sp. SBST2-5^T	*S. thermoviolaceus* NBRC 13905^T	81.75	78.51	25.9	23.6 – 28.4	0.1677	0.01	0.4
	S. emeiensis CGMCC 4.3504^T	83.6	80.7	27.8	25.4 – 30.3	0.1550	0.04	0.7
	S. griseoflavus JCM 4479^T	84.3	82.3	28.6	26.2 – 31.1	0.1501	0.06	0.0
	S. ghanaensis ATCC 14672^T	84.9	82.8	30.0	27.6 – 32.5	0.1423	0.10	2.4

Table S3 Differential phenotypic characteristics of strain SBST2-5^T and the closest phylogenetically relative, and *Streptomyces thermoviolaceus* NBRC 13905^T.

Strains: 1, strain SBST2-5^T; 2, *S. thermoviolaceus* NBRC 13905^T; 3, *S. emeiensis* CGMCC 4.3504^T; 4, *S. griseoflavus* JCM 4479^T; 5, *S. ghanaensis* ATCC 14672^T.

a, the results were determined in this study. Data were taken from: b, Sun *et al*.[25], c, Goodfellow *et al*.[26], d, Goodfellow *et al*.[27], e, Tian *et al*.[28]

+, Positive; -, Negative; w, Weakly positive; ND, not determined.

Characteristics	1[a]	2[a]	3[b]	4[c]	5[d,e]
The colour of aerial mycelium on ISP 2 medium (14 days)	Grayish white	Yellowish white	Dark green	Grey	Grey
Maximum NaCl tolerance (%w/v)	6	1	5	ND	9
Temperature range for growth (°C)	20–55	30–55	15-40	ND	10-50
The pH range for growth	5–10	6–8	5.5-9.5	ND	5-9
Milk peptonization	-	+	+	ND	-
Nitrate reduction	+	-	+	ND	-
Carbon utilization (1.0% w/v):					
L–arabinose	+	-	+	-	-
D–cellobiose	w	-	ND	w	+
D–galactose	+	-	-	-	+
Myo–inositol	+	-	+	+	+
Inulin	+	-	-	ND	ND
D–mannitol	+	-	+	+	ND
D–mannose	+	-	ND	-	+
D–melibiose	w	-	+	-	+
D–raffinose	w	-	-	-	-
Sucrose	+	-	-	-	-
D–trehalose	+	-	ND	ND	+
Xylitol	+	-	ND	ND	-
D-xylose	+	-	w	+	+
Nitrogen utilization (1.0% w/v):					
DL–2–aminobutyric acid	w	-	ND	ND	-
L–cysteine	-	+	-	-	+
L–histidine	+	+	ND	ND	+
L–methionine	+	-	ND	w	-
L–phenylalanine	+	-	+	+	+
Decomposition (1.0% w/v) of:					
Hypoxanthine	+	-	ND	ND	+
L–tyrosine	+	-	ND	ND	+
Enzyme activity:					
α–chymotrypsin	-	w	w	ND	-
Esterase lipase (C8)	-	w	-	ND	+
β–galactosidase	+	-	+	ND	+
α–mannosidase	-	+	+	ND	w
Naphthol–AS–BI–phosphohydrolase	-	+	+	ND	-
Trypsin	-	+	+	ND	w

Table S4 General features of the genome sequences of SBST2-5 and the closest phylogenetically relatives, *S. thermoviolaceus* NBRC 13905^T, *S. emeiensis* CGMCC 4.3504^T, *S. griseoflavus* JCM 4479^T, and *S. ghanaensis* ATCC 14672^T.

**Features**	***Streptomyces* sp. SBST2-5**	***S. thermoviolaceus* NBRC 13905^T**	***S. emeiensis* CGMCC 4.3504^T**	***S. griseoflavus* JCM 4479^T**	***S. ghanaensis* ATCC 14672^T**
**Bioproject**	PRJNA613987	PRJNA613987	PRJEB15726	PRJDB10510	PRJNA32145
**Accession no.**	JAATEM000000000	JAATEL000000000	FMZK00000000	BMUC00000000	ABYA00000000
**Genome coverage**	200X	150X	91X	165X	11.6X
**N50**	166,826	183,806	329,446	115,138	24,170
**Number of Contigs**	100	85	32	151	616
**Genome size (Mb)**	6.52	5.89	7.13	7.58	8.51
**DNA G+C content (%)**	72.2	72.6	72.9	72.2	69.8
**Number of genes**	5,820	5,156	6,259	6,916	7,807
**Protein coding genes**	5,516	4,870	5,952	6,643	7,044
**Number of RNAs**	76	77	79	78	82
**rRNA**	3	4	12	4	6
**tRNA**	70	70	64	71	73
**Other RNA**	3	3	3	3	3
**Pseudogene**	228	209	228	195	681

Table S5 Biosynthetic gene clusters (>50% similarity with known bioclusters) found in antiSMASH for the *Streptomyces* strains in this study.

Cluster	Type	From	To	Most similar known cluster to	MIBiG BGC-ID
***Streptomyces* sp. SBST2-5^T**					
Region 1.1	T2PKS	45,893	118,405	Spore pigment (83% of genes show similarity)	BGC0000271
Region 4.1	NRPS-independent-siderophore	127,121	138,893	Desferrioxamin B (83% of genes show similarity)	BGC0000940
Region 13.1	Terpene	34,043	59,859	Isorenieratene (85% of genes show similarity)	BGC0000664
Region 18.1	Terpene	83,943	105,028	Albaflavenone (100% of genes show similarity)	BGC0000660
Region 34.1	Ectoine	81,275	91,673	Ectoine (100% of genes show similarity)	BGC0000853
Region 43.1	NRPS, T1PKS	1	31,800	Tirandamycin (86% of genes show similarity)	BGC0001052
Region 50.1	Terpene	1	13,269	Hopene (53% of genes show similarity)	BGC0000663
Region 84.1	Terpene	47,860	70,046	Geosmin (100% of genes show similarity)	BGC0001181
***S. thermoviolaceus* NBRC 13905^T**					
Region 4.2	Terpene	54,382	79,713	Carotenoid (63% of genes show similarity)	BGC0000633
Region 5.1	T2PKS	51,745	124,248	Spore pigment (83% of genes show similarity)	BGC0000271
Region 12.3	Terpene	270,077	292,284	Geosmin (100% of genes show similarity)	BGC0001181
Region 14.1	T2PKS	32,816	105,325	Granaticin (54% of genes show similarity)	BGC0000227
Region 16.1	Terpene	1	18,492	Hopene (53% of genes show similarity)	BGC0000663
Region 30.1	NRPS-independent-siderophore	33,197	44,966	Desferrioxamin B (83% of genes show similarity)	BGC0000940
Region 62.1	Ectoine	67,103	77,507	Ectoine (100% of genes show similarity)	BGC0000853
Region 64.1	T3PKS	98,257	139,315	Flaviolin (100% of genes show similarity)	BGC0002127

Table S6 Identified ORFs, putative gene functions, and perspective homologs in the tirandamycin (*tam*) biosynthetic gene cluster in the genome of strain SBST2-5^T.

Gene in SBST2-5^T	ORFs* from *tam* Gene Cluster		Putative Product and Their Accession number (% Identity)**
	Gene	% Identity/Coverage	
Orf1			Acyl transferase domain-containing protein of *Streptomyces* sp. SLBN-118 (91.8%) (A0A542QLU6)
Orf2	*tamAIII*	95.4/100	Acyl transferase domain-containing protein of *Streptomyces* sp. SLBN-118 (95.4%) (A0A542QLX2)
Orf3	*tamB*	98.8/100	Surfactin synthase thioesterase subunit of *Streptomyces* sp. SLBN-118 (97.7%) (A0A542QLS7)
Orf4			CUB domain-containing protein of *Cudoniella acicularis* (42.9%) (A0A8H4R6R7)
Orf5	*tamC*	97.1/100	Thioesterase domain-containing protein of *Streptomyces* sp. SLBN-118 (97.1%) (A0A542QLS8)
Orf6	*tamD*	97.8/100	Amino acid adenylation domain-containing protein of *Streptomyces* sp. SLBN-118 (96.3%) (A0A542QLT6)
Orf7	*tamE*	97.8/100	Glycosyl hydrolase family 16 of *Streptomyces* sp. SLBN-118 (97.5%) (A0A542QLS5)
Orf8	*tamF*	97.7/100	Prenyltransferase/squalene oxidase-like repeat protein of *Streptomyces* sp. SLBN-118 (94.6%) (A0A542QLU2)
Orf9	*tamG*	98.7/97	DNA helicase IV of *Streptomyces* sp. SLBN-118 (97.3%) (A0A542QLT7)
Orf10			Protein EXORDIUM-like 2 of *Selaginella moellendorffii* (Spikemoss) (53.3%) (D8QR28)
Orf11	*tamH*	92.9/100	Regulatory *LuxR* family protein of *Streptomyces* sp. SLBN-118 (97.8%) (A0A542QLU3)
Orf12	*tamI*	98.3/99	Cytochrome P450 of *Streptomyces* sp. SLBN-118 (99.3%) (A0A542QLU4)
Orf13	*tamJ*	98.6/100	*EmrB*/*QacA* subfamily drug resistance transporter of *Streptomyces* sp. SLBN-118 (98.4%) (A0A542QLV7)
Orf14			SnoaL-like domain-containing protein of *Nonomuraea aridisoli* (58.1%) (A0A2W2D9A1)
Orf15	*tamK*	99.5/100	*TetR* family transcriptional regulator of *Streptomyces* sp. SLBN-118 (98.1%) (A0A542QLY5)
Orf16			*PaiB* family negative transcriptional regulator of *Streptomyces* sp. SLBN-118 (97.8%) (A0A542QLT9)
Orf17			2-polyprenyl-6-methoxyphenol hydroxylase-like FAD-dependent oxidoreductase of *Streptomyces* sp. SLBN-118 (94.3%) (A0A542QLU0)
Orf18			Phosphatase *PAP2* family protein of *Streptomyces palmae* (64.8%) (A0A4Z0HFL3)
Orf19			*UbiE*/*COQ5* family methyltransferase of *Streptomyces griseoflavus* Tu4000 (82.1%) (D9XKJ8)

*Open reading frame compared to the described *tam* cluster from *Streptomyces* sp. 307-9.

**Best match found by UniProtKB Protein-Protein BLAST.

Table S7. ^{1}H (500 MHz) and ^{13}C NMR (125 MHz) spectral data of (−)-Tirandamycin B (**1**) in $CDCl_3$.

Position	Tirandamycin B (**1**) ^{1}H NMR δ_H, mult. (*J in Hz*)	^{13}C NMR δ_C, Type
1		175.1, C
2	7.16, d (15.7)	116.8, CH
3	7.57, d (15.7)	149.6, CH
4		135.0, C
5	6.19, d (9.9)	143.3, CH
6	2.86, ttd (9.9, 6.9, 1.9)	34.4(7)[a], CH
7	3.66, dd (11.5, 1.9)	77.2, CH
8	1.99, dt (10.9, 5.8)	34.5[a], CH
9	4.05, d (6.1)	78.7, CH
10		201.4, C
11	3.69, s	56.9, CH
12		59.3, C
13		95.9, C
14	1.58, s	23.3, CH_3
15	1.92, s	12.3, CH_3
16	1.13, d (6.9)	16.9, CH_3
17	0.73, d (7.1)	11.4, CH_3
18	3.97, d (12.7) / 4.01, d (12.7)	58.1, CH_2
1′(NH)		
2′		176.4, C
3′		100.1, C
4′		192.5, C
5′	3.83, s	51.6, CH_2
OH	5.94, s	

[a] interchangeable

Table S8. Biological activity of compound 1 (TAM B).

**Compound**	**Anti-BACE1 at 2 μg/ml (% inhibition)**	**Anti-BACE1 at 20 μg/ml (% inhibition)**	**Anti-BACE1 at 200 μg/ml (% inhibition)**	**Neuroprotective ability[a] at 1 ng/ml (% cell viability)**	**Anti-AchE[b] (% inhibition)**	**DPPH radical scavenging activity[c] IC_{50} (μg/mL)**	**Cytotoxicity against Vero cell (IC_{50}, μg/mL)[d]**
Compound 1 (TAM B)	37.73 ± 6.25	68.69 ± 8.84	97.37 ± 6.25	116.53 ± 11.60	5.36 ± 1.29	166.30 ± 8.9	>1000.0
Quercetin (2 μg/ml) (positive control)	36.65 ± 10.14						
Quercetin (20 μg/ml) (positive control)		67.56 ± 9.40					
Quercetin (200 μg/ml) (positive control)			99.81 ± 7.51				
Quercetin (1 ng/ml) (positive control)				77.01 ± 14.74			
Galantamine (1 μg/ml) (positive control)					75.98 ± 1.65		
BHT (positive control)						57.73 ± 2.4	
Ellipticine (positive control)							1.67

Values are mean ± SEM (n=3).
[a] % Cell viability for tested compounds versus control cells was calculated by the absorbance at 450 nm from three independent experiments and each experiment was run in triplicate.

[b] Maximum tested concentration was at 250 μg/mL.
[c,d] Maximum tested concentration was at 1000 μg/mL.

Table S9. Ten-ranking score of Autodock Vina docking poses of atabecestat and TAM B into the binding site of human BACE1.

**Pose number**	**Vina docking score (kcal/mol)**	
	**Atabecestat**	**Tirandamycin B**
1	-8.0	-8.3
2	-7.6	-8.1
3	-7.6	-7.9
4	-7.4	-7.7
5	-7.3	-7.7
6	-7.1	-7.7
7	-7.0	-7.7
8	-6.9	-7.6
9	-6.9	-7.6
10	-6.6	-7.5
11	-6.5	-7.5
12	-6.5	-7.5
13	-6.5	-7.4
14	-6.5	-7.3
15	-6.5	-7.2
16	-6.4	-7.1
17	-6.4	-7.1
18	-6.4	-7.0
19	-6.3	-6.9
20	-6.3	-6.8

Figure S1 Colonial appearance of strain SBST2-5^{T}.

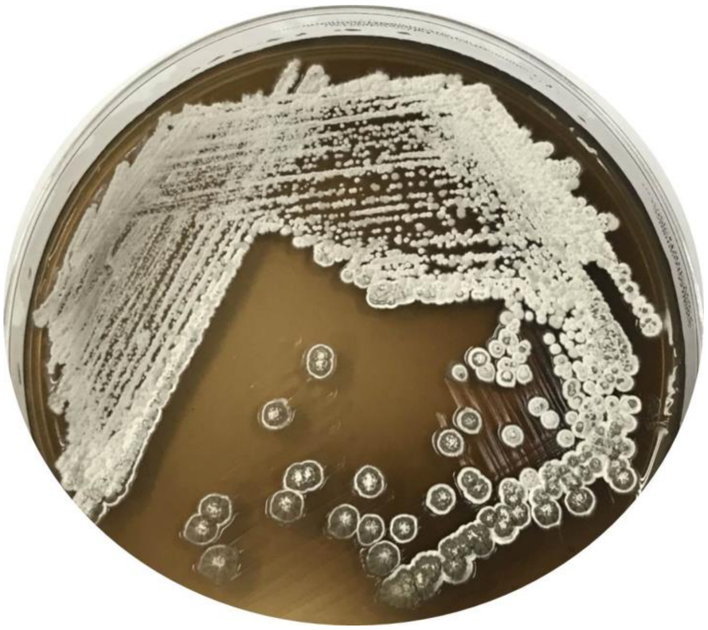

Figure S2 Whole-cell sugar pattern of strain SBST2-5^{T}.

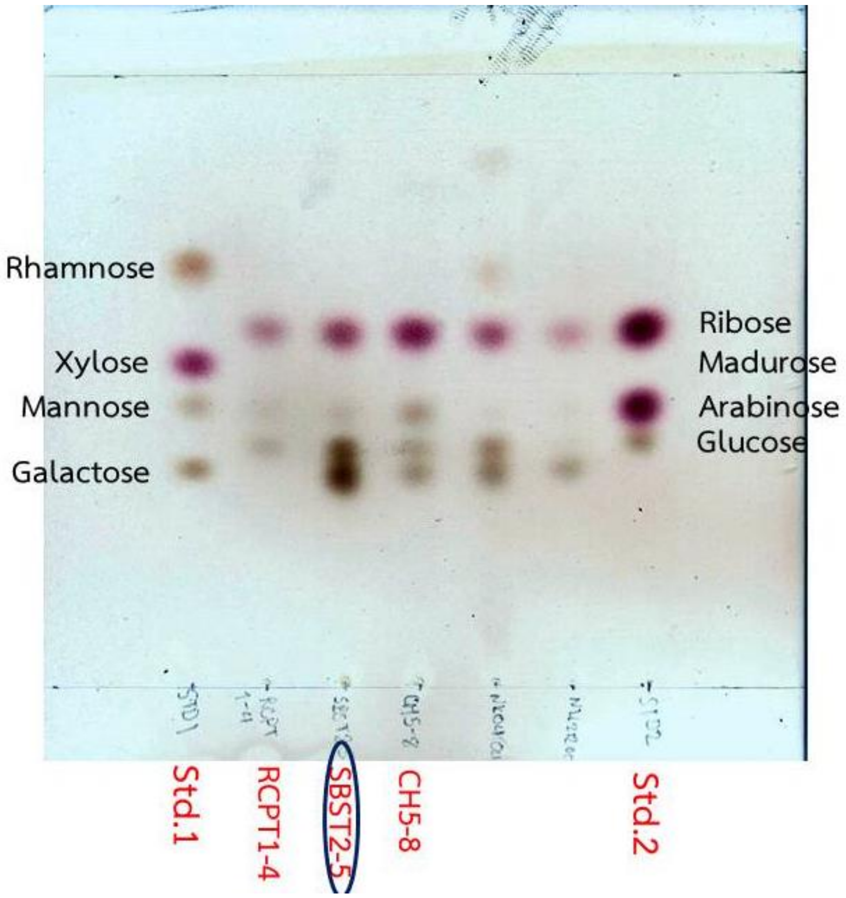

Figure S3 Polar lipid appearing on the two-dimensional thin-layer chromatograms of strain SBST2-5^{T}. (A) Phosphomolybdic acid's TLC chromatogram; (B) Molybdenum blue spray's TLC chromatogram; (C) Anisaldehyde's TLC chromatogram; (D) Ninhydrin's TLC chromatogram; (E) Dragendorff's TLC chromatogram.

Abbreviation: diphosphatidylglycerol (DPG), ninhydrin-positive lipid (NPL), phosphatidylethanolamine (PE), phosphatidylinositol (PI), phosphatidylinositol mannoside (PIM), and unidentified phospholipid (PL). The solvent systems used for development are as follows:

The 1st solvent system : Chloroform:MeOH:Water (65:25:4)

The 2nd solvent system : Chloroform:Acetic acid:MeOH:Water (40:7.5:6:2)

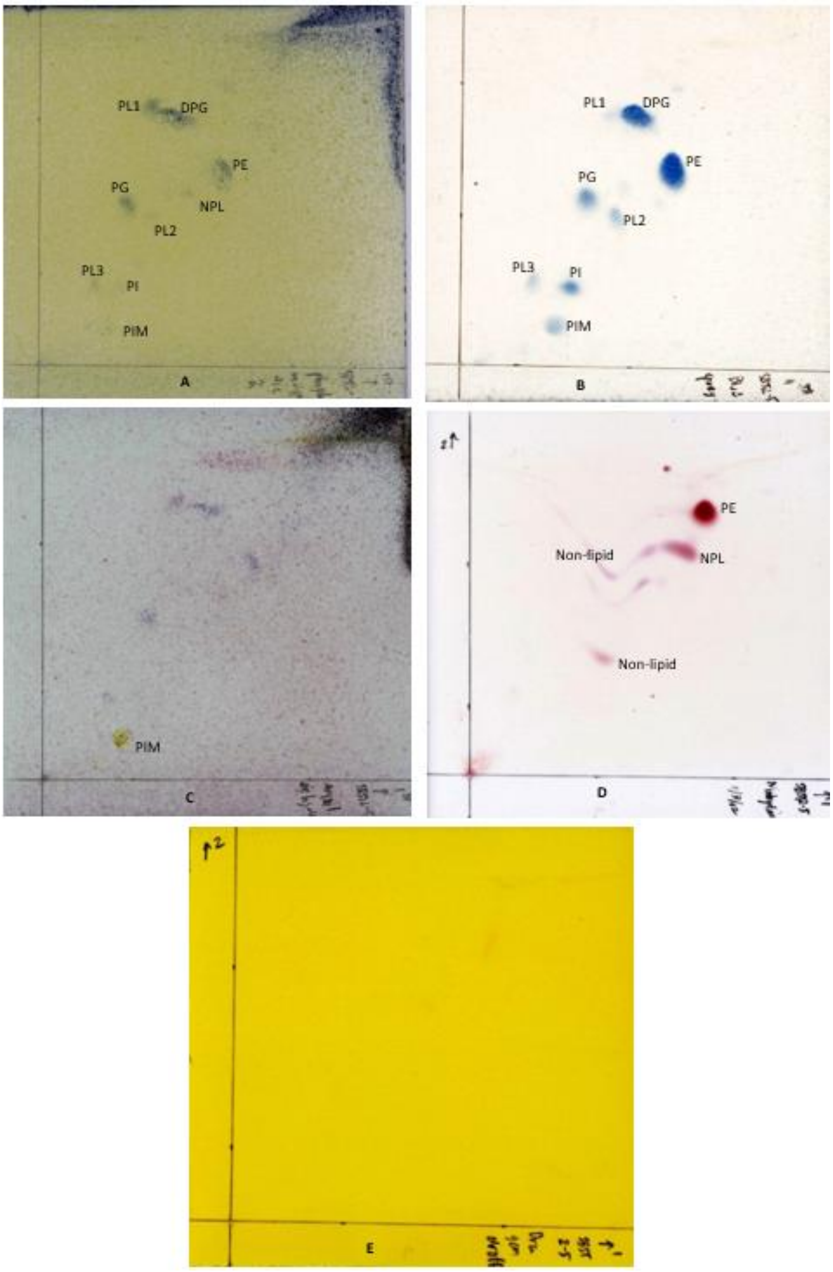

Figure S4 Maximum-Likelihood (ML) phylogenetic tree based on 16S rRNA gene sequences comparing strain SBST2-5^{T} to *Streptomyces* species. *Micromonospora carbonacea* DSM 43168^{T} was used as the out-group. The numbers on the branches indicate the percentage bootstrap values of 1,000 replicates. Bar, 0.02 substitutions per nucleotide position.

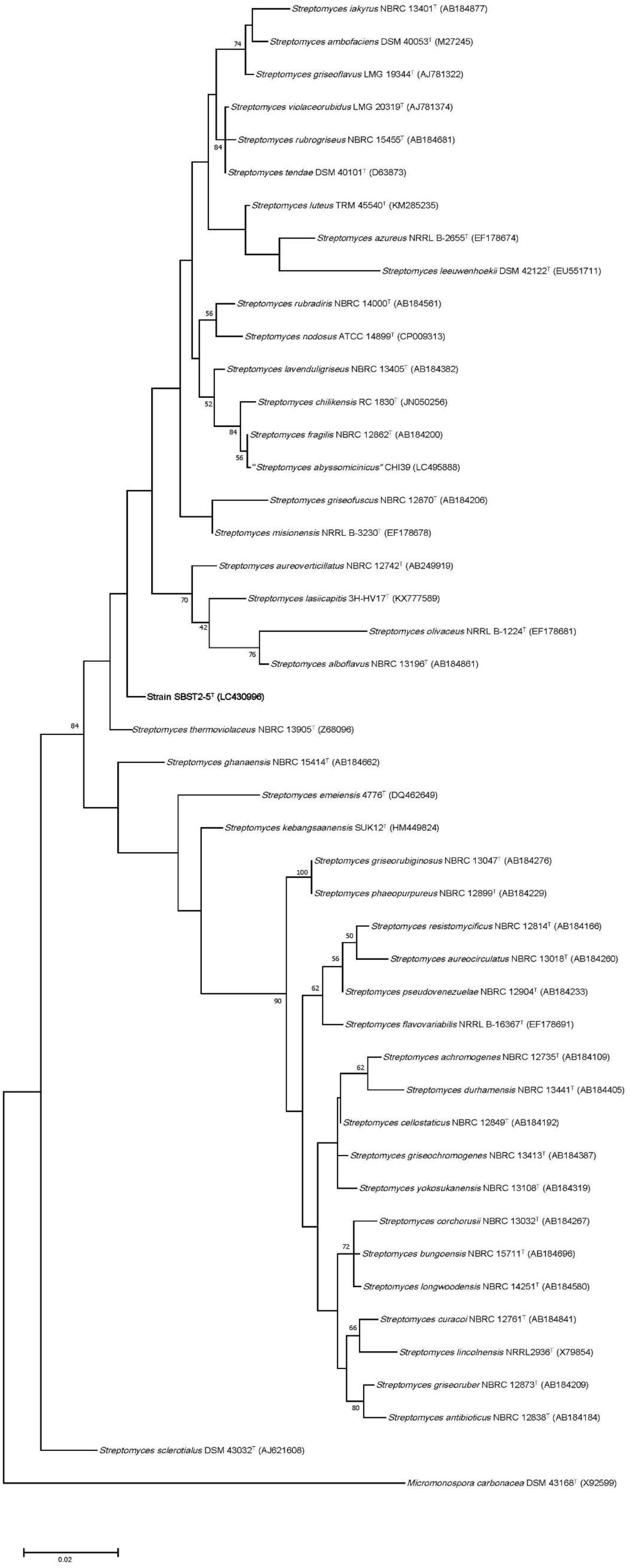

Figure S5 Neighbor-joining (NJ) phylogenetic tree based on 16S rRNA gene sequences comparing strain SBST2-5^T to *Streptomyces* species. *Micromonospora carbonacea* DSM 43168^T was used as the out-group. The numbers on the branches indicate the percentage bootstrap values of 1,000 replicates. Bar, 0.01 substitutions per nucleotide position.

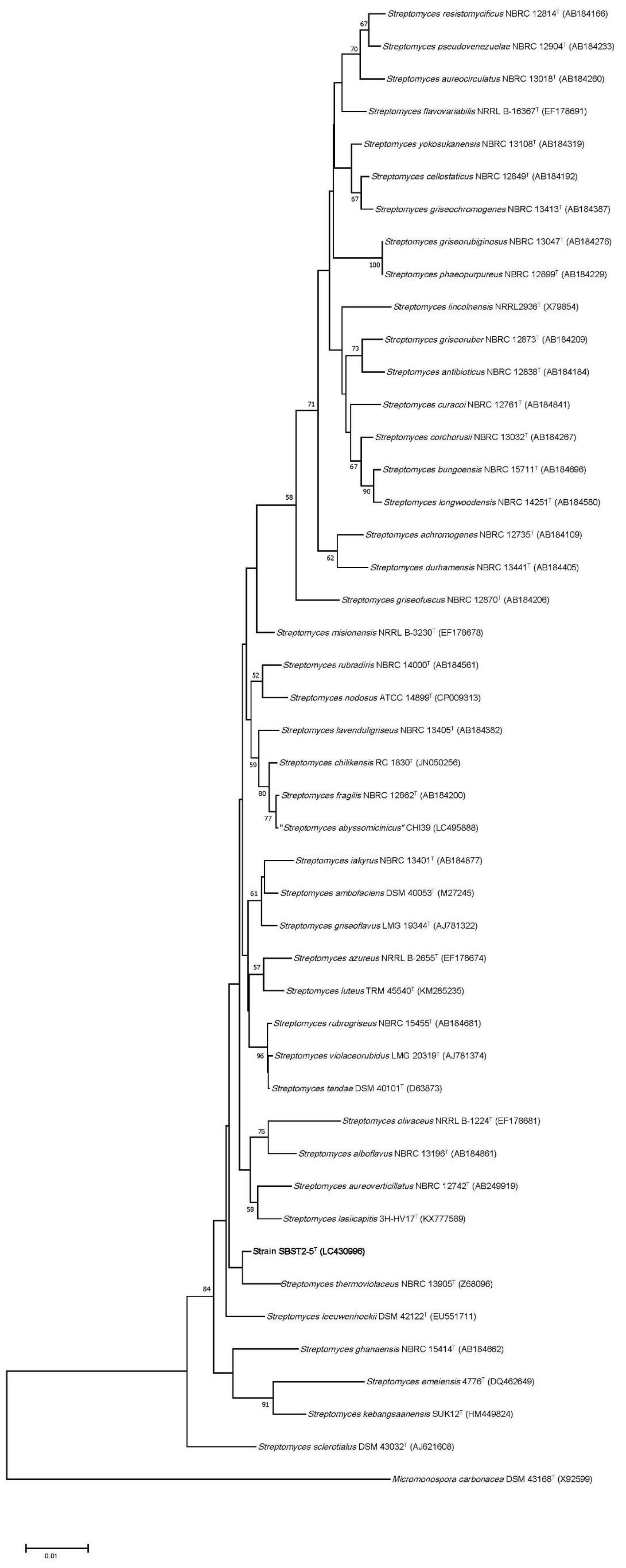

Figure S6 Biosynthetic gene clusters presented in the genomes of *Streptomyces* sp. SBST2-5^T and its close relative using antiSMASH 7 beta.

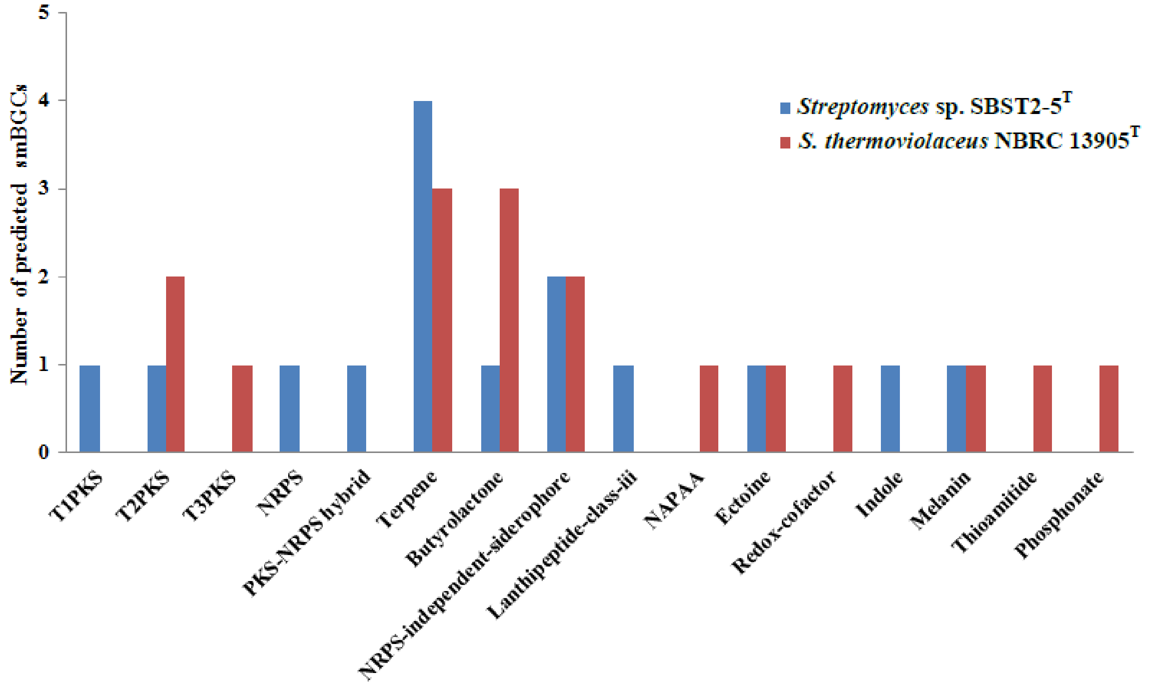

Figure S7 Comparison of the tirandamycin biosynthetic gene clusters from *Streptomyces* sp. 307-9 and *Streptomyces* sp. SBST2-5^{T}.

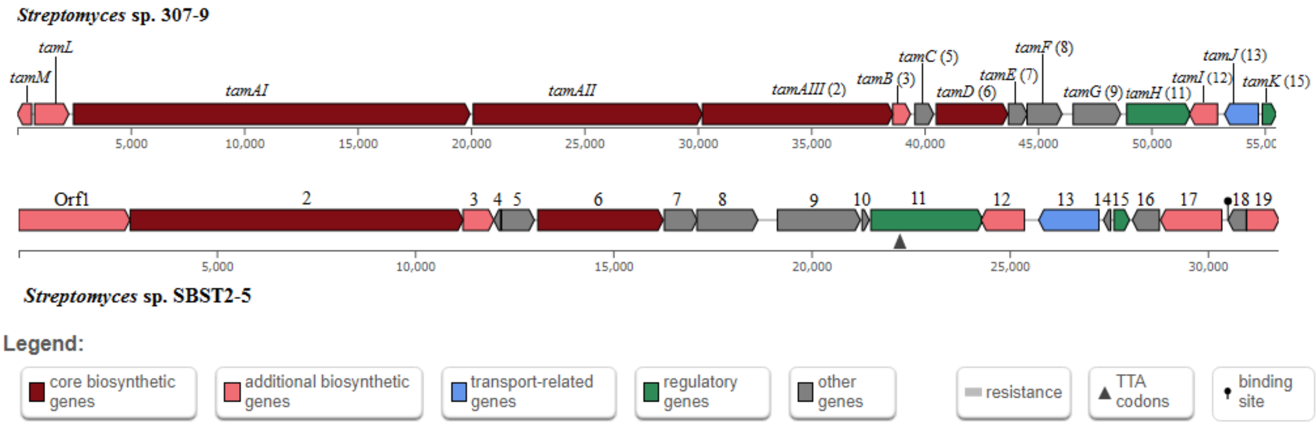

*(−)-Tirandamycin B (**1**)*: pale yellow solid; $[\alpha]^{25}_{D}$ −30.19 (*c* 0.34, MeOH) and $[\alpha]^{27}_{D}$ −14.64 (*c* 0.16, EtOH); UV (MeOH) λmax (log ε) 202 (4.03), 250 (3.75), 290 (3.82), 339 (4.10); HRESIMS *m/z* 432.1662 $[M–H]^{–}$ (calcd for $C_{22}H_{26}NO_8$, 432.1664).

Figure S8 ^{1}H NMR spectrum of compound **1** in $CDCl_3$ (400 MHz).

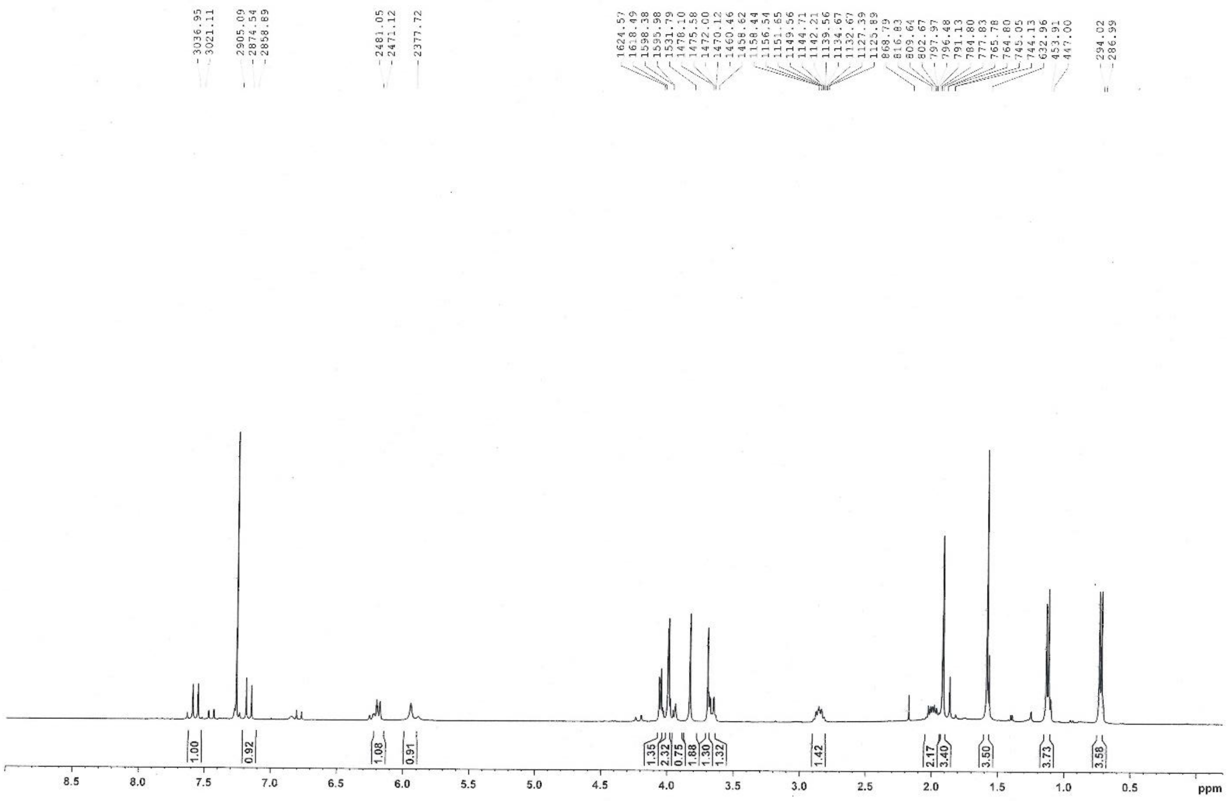

Figure S9 ^{13}C NMR spectrum of compound **1** in $CDCl_3$ (100 MHz).

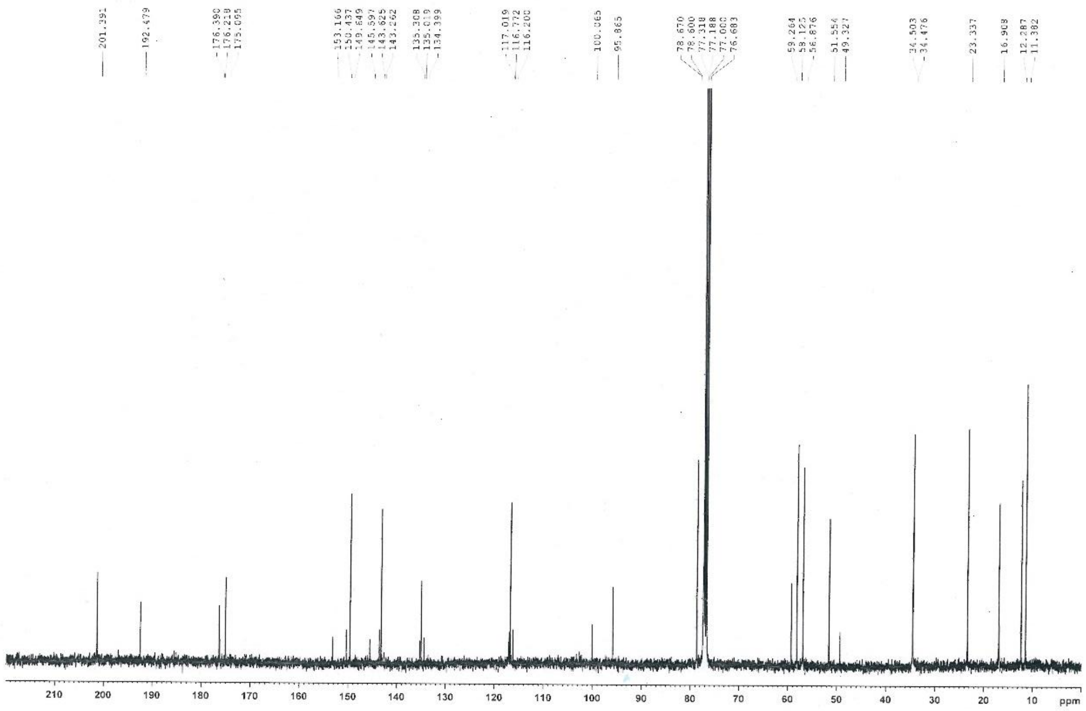

Figure S10 HRESIMS spectrum of compound **1.**

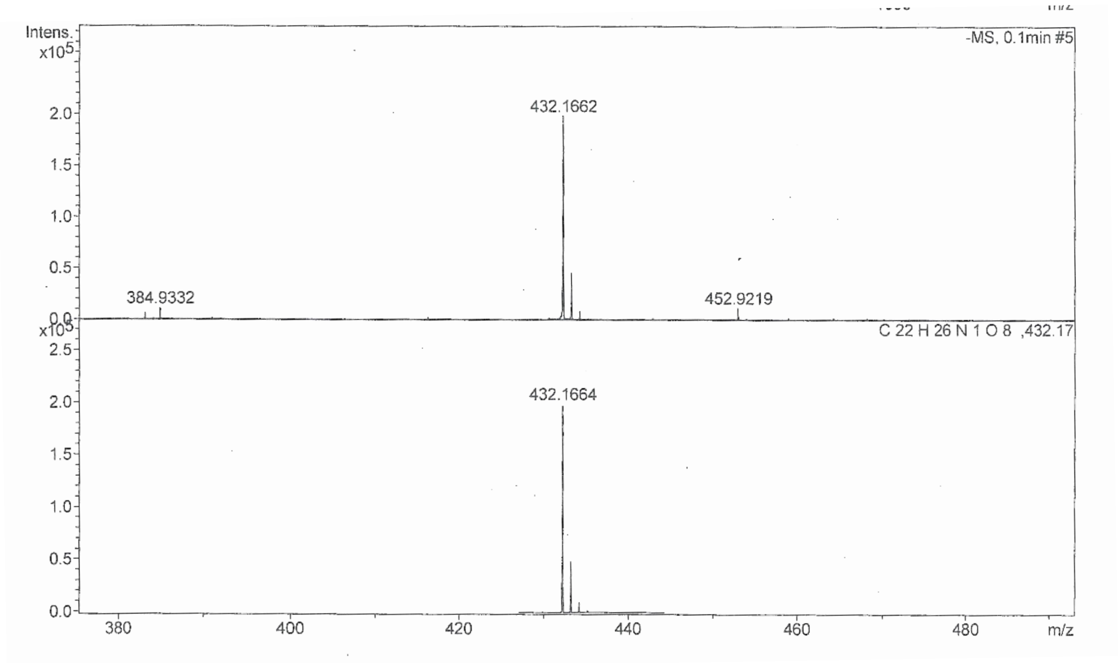

Figure S11 Viability of P19-derived neuron treated with the compound 1 at various concentrations of 1-10000 ng/ml. The error bar represented standard error of the mean (SE). The 0.5%DMSO in the medium was used as a control representing no effects on the neuronal viability (100.02 ± 1.40% neuron viability) of the solvent.

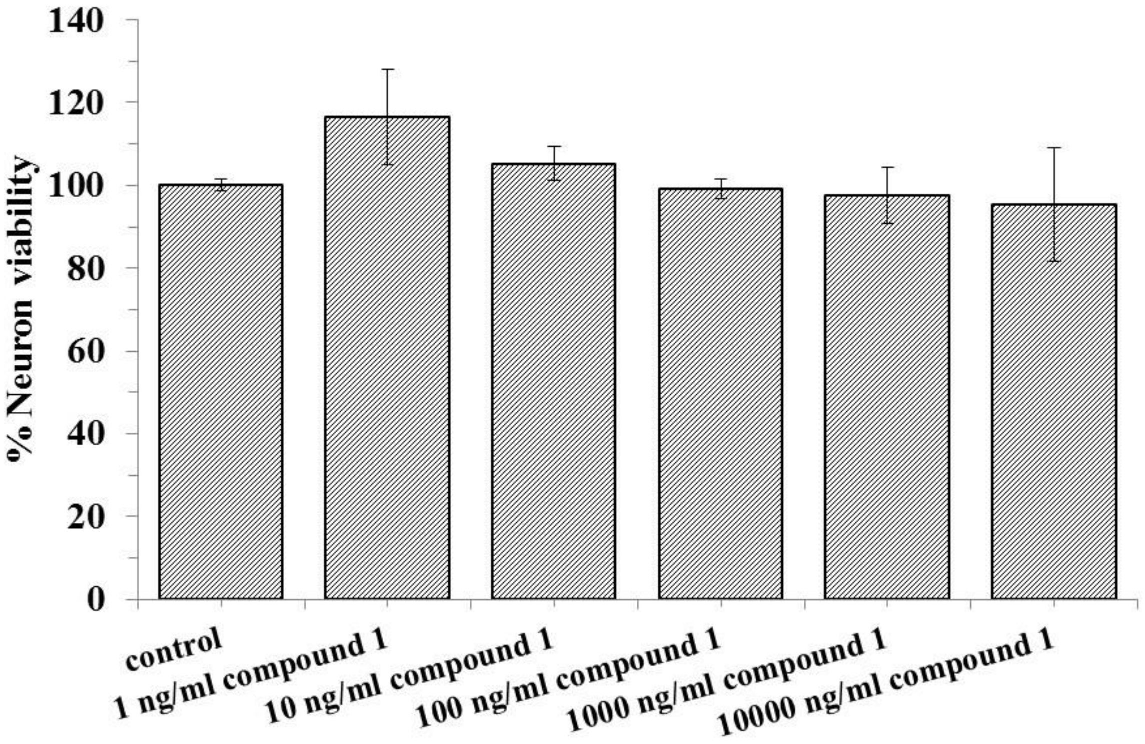

Figure S12 Neuroprotective ability at 1 ng/ml of compound 1 on P19-derived neuron. The error bar represented the standard error of the mean (SE).

(* $p < 0.05$ when compared to oxidative stress condition produced by serum deprivation)

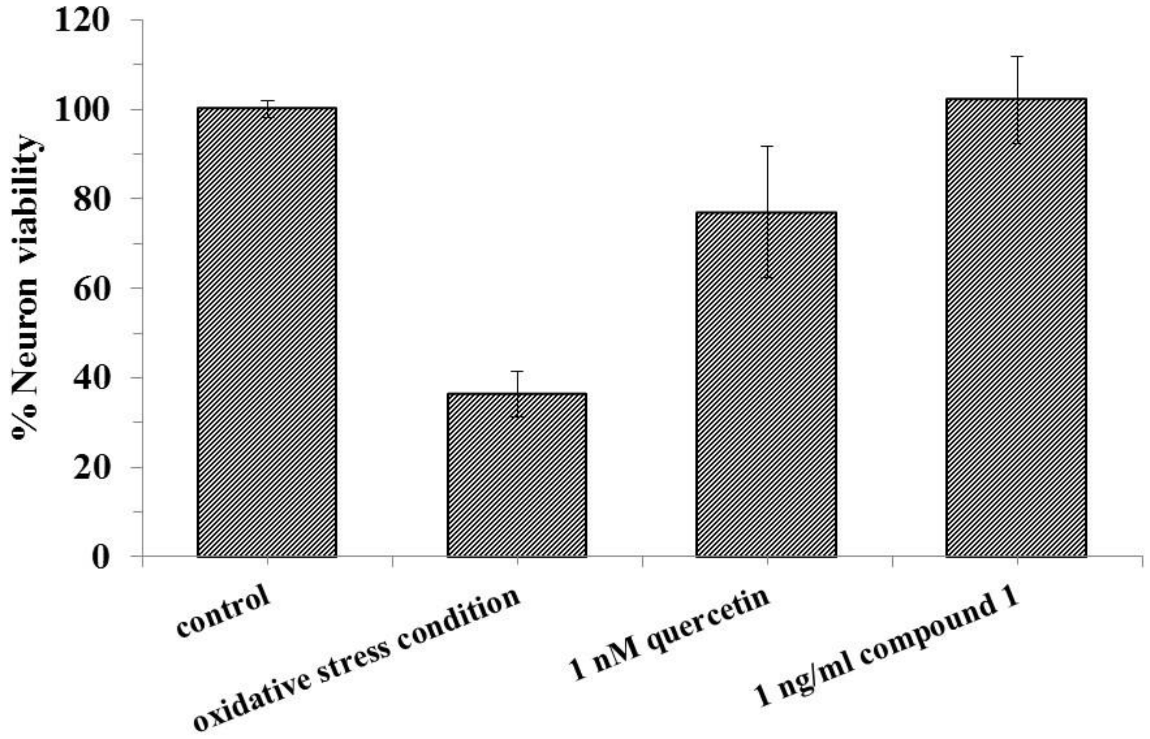

Figure S13 Protective effect of compound 1 against Aβ1-42 induced cell death. All values are represented as mean ± SD of at least three independent experiments.

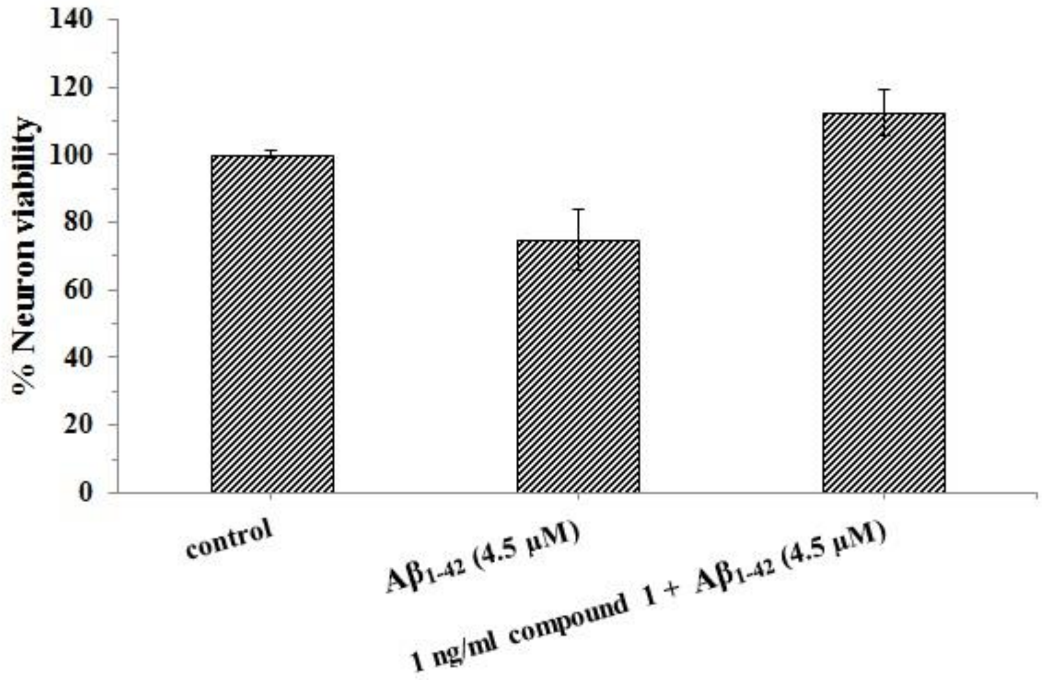

Figure S14 Cytotoxicity on HEK293 cell of compound 1**.**

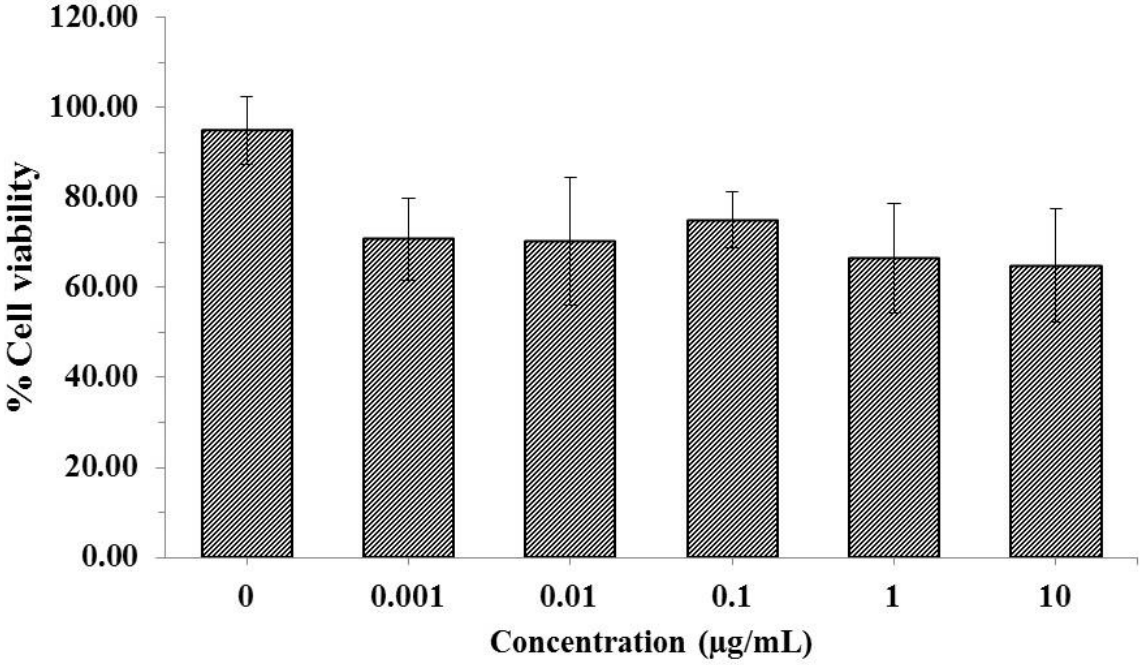

Figure S15 Top-10 ranking poses of atabecestat (A) and TAM B (B) in the binding site of human BACE1 by Autodock Vina.

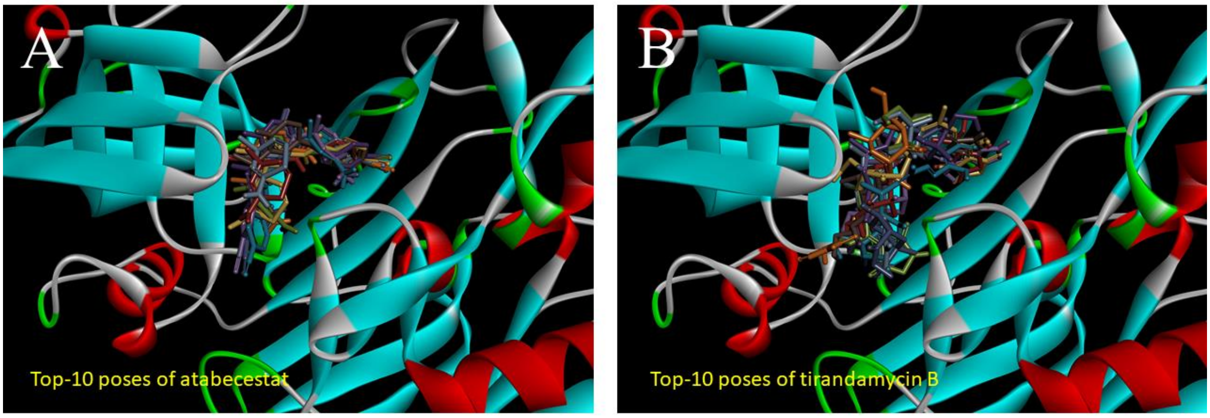

Figure S16 ADMET prediction of TAM B using ADMETlab 2.0 (https://admetmesh.scbdd.com/)

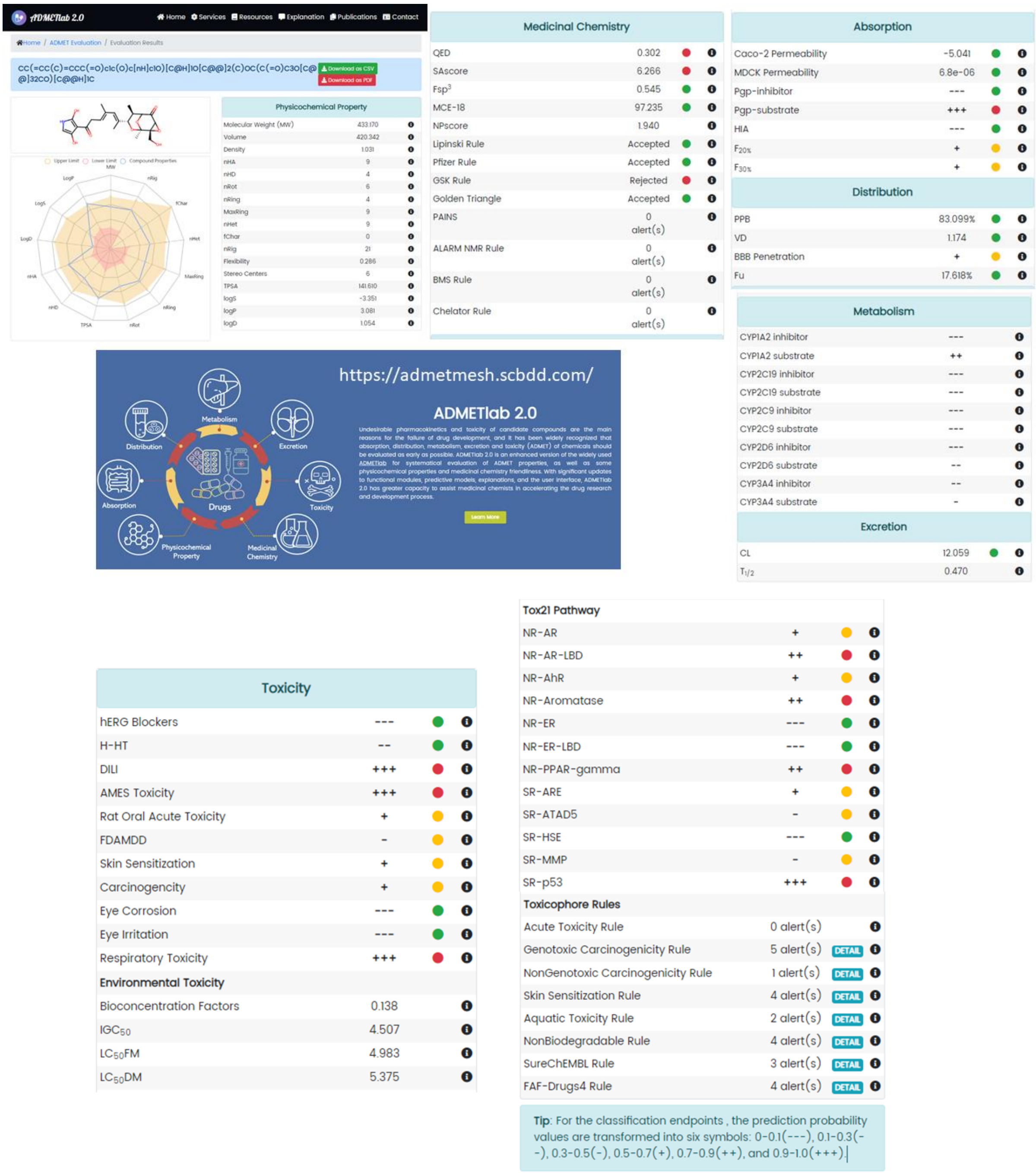

Physicochemical Property	
Molecular Weight (MW)	433.170
Volume	420.342
Density	1.031
nHA	9
nHD	4
nRot	6
nRing	4
MaxRing	9
nHet	9
fChar	0
nRig	21
Flexibility	0.286
Stereo Centers	6
TPSA	141.610
logS	-3.351
logP	3.081
logD	1.054

Medicinal Chemistry	
QED	0.302
SAscore	6.266
Fsp^3	0.545
MCE-18	97.235
NPscore	1.940
Lipinski Rule	Accepted
Pfizer Rule	Accepted
GSK Rule	Rejected
Golden Triangle	Accepted
PAINS	0 alert(s)
ALARM NMR Rule	0 alert(s)
BMS Rule	0 alert(s)
Chelator Rule	0 alert(s)

Absorption	
Caco-2 Permeability	-5.041
MDCK Permeability	6.8e-06
Pgp-inhibitor	---
Pgp-substrate	+++
HIA	---
$F_{20\%}$	+
$F_{30\%}$	+
**Distribution**	
PPB	83.099%
VD	1.174
BBB Penetration	+
Fu	17.618%
**Metabolism**	
CYP1A2 inhibitor	---
CYP1A2 substrate	++
CYP2C19 inhibitor	---
CYP2C19 substrate	---
CYP2C9 inhibitor	---
CYP2C9 substrate	---
CYP2D6 inhibitor	---
CYP2D6 substrate	--
CYP3A4 inhibitor	--
CYP3A4 substrate	-
**Excretion**	
CL	12.059
$T_{1/2}$	0.470

Toxicity	
hERG Blockers	---
H-HT	--
DILI	+++
AMES Toxicity	+++
Rat Oral Acute Toxicity	+
FDAMDD	-
Skin Sensitization	+
Carcinogencity	+
Eye Corrosion	---
Eye Irritation	---
Respiratory Toxicity	+++
**Environmental Toxicity**	
Bioconcentration Factors	0.138
IGC_{50}	4.507
$LC_{50}FM$	4.983
$LC_{50}DM$	5.375

Tox21 Pathway	
NR-AR	+
NR-AR-LBD	++
NR-AhR	+
NR-Aromatase	++
NR-ER	---
NR-ER-LBD	---
NR-PPAR-gamma	++
SR-ARE	+
SR-ATAD5	-
SR-HSE	---
SR-MMP	-
SR-p53	+++
**Toxicophore Rules**	
Acute Toxicity Rule	0 alert(s)
Genotoxic Carcinogenicity Rule	5 alert(s) DETAIL
NonGenotoxic Carcinogenicity Rule	1 alert(s) DETAIL
Skin Sensitization Rule	4 alert(s) DETAIL
Aquatic Toxicity Rule	2 alert(s) DETAIL
NonBiodegradable Rule	4 alert(s) DETAIL
SureChEMBL Rule	3 alert(s) DETAIL
FAF-Drugs4 Rule	4 alert(s) DETAIL

Tip: For the classification endpoints, the prediction probability values are transformed into six symbols: 0-0.1(---), 0.1-0.3(--), 0.3-0.5(-), 0.5-0.7(+), 0.7-0.9(++), and 0.9-1.0(+++).
